# Associations between the 2022 global mpox outbreak and multifaceted factors: A multi-geographical retrospective study

**DOI:** 10.1016/j.onehlt.2025.101224

**Published:** 2025-09-24

**Authors:** Yuxi Ge, Yifei Wang, Ziqin Zhou, Zhirui Zhang, Yunyu Tian, Yun Feng, Peiyi Wu, Yuxin Wang, Ziyan Liu, Bingying Li, Zengmiao Wang

**Affiliations:** a Beijing Key Laboratory of Surveillance, Early Warning and Pathogen Research on Emerging Infectious Diseases, State Key Laboratory of Remote Sensing and Digital Earth, Center for Global Change and Public Health, Faculty of Geographical Science, Beijing Normal University, Beijing, China; b School of Statistics, Beijing Normal University, Beijing, China; c National Key Laboratory of Intelligent Tracking and Forecasting for Infectious Diseases, Beijing, China

**Keywords:** Mpox, Outbreak probability, One health, Long-term

## Abstract

Unlike prior travel-related mpox cases, the 2022 outbreak of mpox virus (MPXV) clade IIb outside endemic regions posed a significant global health threat. Despite growing recognition of the One Health relevance of mpox, the factors driving this unprecedented outbreak and their quantitative effects remain not fully understood. This study aims to identify key factors across various geographical scales to inform future mpox mitigation policies within One Health framework. We built logistic regression models to assess the association of 18 covariates—including socioeconomic, demographic, and human behaviors (e.g., urbanization, sexual behavior, immunity, mobility and contact intensity) —with mpox outbreak probability in the United States, England, Brazil, and globally. We also examined temporal trends over the past decade. Our analysis revealed positive associations between mpox cases and urbanization rates (United States: *R* = 0.43; England: *R* = 0.25; Brazil: *R* = 0.52; all *P* < 0.05) and the proportion of lesbian, gay, bisexual, and transgender (LGBT) individuals (United States: *R* = 0.39; England: *R* = 0.72; both *P* < 0.05; Brazil: *R* = 0.26, *P* = 0.18). Conversely, smallpox vaccination coverage showed a negative association with mpox cases (United States: *R* = -0.25, *P* = 0.08; England: *R* = -0.52). Similar trends were observed globally. Mpox outbreak probability increased globally over the past decade. Our findings highlight the role of long-term human behavior changes in MPXV clade IIb outbreaks. From a One Health perspective, these results suggest that ongoing attention to behavioral factors, alongside ecological and social contexts, may help improve understanding and prediction of outbreak dynamics.

## 1 Introduction

Mpox, a zoonotic disease caused by mpox virus (MPXV), was initially detected in a human case in 1970 in the Democratic Republic of the Congo [1,2]. Although mpox infection has become established in West and Central Africa since then, imported travel-related cases has occasionally been reported in non-endemic regions, such as the United States [3], the United Kingdom [4], Israel [5], and Singapore [6]. A notable shift occurred in May 2022, when a large-scale international outbreak of human mpox cases caused by MPXV clade IIb emerged, characterized by transmission through sexual contact and raising significant global public health concerns [7]. In response to the escalating threat, the World Health Organization (WHO) declared the mpox outbreak a public health emergency of international concern (PHEIC) on July 23, 2022 [8]. This status was lifted 10 months later due to a substantial drop in global mpox case numbers [9]. Nonetheless, mpox cases began climbing again in early 2023, particularly in several Asian countries such as Japan [10] and China [11]. Alarmingly, a distinct MPXV strain—named clade Ib—was linked to sexually transmitted infections starting in September 2023 [12]. This strain triggered outbreaks in previously unaffected areas of the Democratic Republic of the Congo and spread to adjacent countries where mpox had not been reported before [13]. With this renewed spread, the WHO designated mpox outbreak as PHEIC again on August 14, 2024 [14]. As a zoonotic pathogen influenced by the interactions of human, animal, and environmental factors, mpox underscores the importance of adopting a One Health perspective. Understanding factors that drove the 2022 outbreak is therefore crucial for developing effective strategies to prevent and control the spread of clade IIb and the emerging clade Ib within a One Health framework.

Recent studies point to potential genetic alterations in MPXV as contributing factors in 2022 global outbreak [15]. A novel phylogenetic lineage, termed B.1, was identified as the dominated variant during the global surge in 2022 [16]. This lineage forms a distinct evolutionary branch, indicating accelerated viral evolution [17]. Additionally, genomic analysis has also identified APOBEC3-related mutations, which imply the ongoing adaptation to human hosts [16,17]. Although the viral genetic traits likely contributed to the transmission of mpox during the outbreak [17,18], they are insufficient to fully explain the outbreak. A more comprehensive investigation that incorporates host, behavioral, and environmental factors framed within a One Health approach is essential to uncover the underlying drivers of the outbreak.

During the 2022 MPXV clade IIb outbreak, men who have sex with men (MSM) constituted the majority of cases, with sexual transmission identified as the key pathway [19]. Retrospective study detecting MPXV in the testes of nonhuman primate supports the hypothesis of sexual transmission in humans [20]. Moreover, contact tracing data suggest that the virus may spread through densely interconnected sexual networks [18,21]. These findings highlight the significant influence of human behavior in driving mpox spread during this period [22]. Mathematical modeling has demonstrated that the continued expansion of mpox among MSM may be due to the highly skewed distribution of sexual partnership at the individual level [23]. Despite these insights, the link between the change of human sexual behavior at the population level and the mpox outbreak has not been quantified.

Other contributing factor related to the mpox outbreak may be the cessation of smallpox immunization programs after 1980, which likely increased overall population vulnerability to MPXV infection [24]. A previous study has also suggested that urbanization is associated with the mpox outbreaks in an endemic region [24]. Furthermore, earlier studies have demonstrated that international travel arrivals are related to the outbreak of mpox at the country level [25]. While these studies provided valuable insights, a multi-scale, population-level analysis of how human behavioral, demographic and environmental dimensions jointly influence mpox transmission is still lacking. Addressing this gap within a One Health framework will enhance our understanding of outbreak dynamic and support future pandemic management.

In this study, we investigated how various factors may have contributed to the mpox outbreak in 2022 across non-endemic settings. We collected data spanning socioeconomic conditions, demographic structures, and human behavior indicators across both national and global scales. Our research focused on England (the first to report mpox cases in 2022 [7]), the United States (which recorded the highest number of mpox cases globally [26]), Brazil (the most affected developing country [26]), as well as global patterns. By applying multiple logistic regression models, we identified the factors related to 2022 mpox outbreak and assessed the probability of mpox outbreaks. Our One Health–guided analysis offers new insights into the multifactorial drivers of the 2022 epidemic and inform strategies for addressing the current clade Ib mpox outbreak, which shares similar sexual transmission characteristics.

## 2 Methods

### 2.1 Study design

In this retrospective observational analysis, we applied both the simple and multiple logistic regression model to quantify the association between socioeconomic (e.g., gross domestic product (GDP), public budget expenditure), demographic (e.g., urbanization rate), and human behavior factors (e.g., smallpox vaccination coverage, sexual behavior, nighttime light index, and mobility data) and the outbreak of mpox in 2022. Comparative assessments were conducted to evaluate outbreak probability in 2022 relative to previous years, at multiple geographic resolutions: U.S. states, England's upper tier local authority (UTLA), Brazilian states, and globally at the national level.

### 2.2 Data collection

We collected the epidemiological data and various factors data (included 18 variables, covering three aspects: socioeconomic, demographic, and human behavior) potentially related to mpox outbreak for 50 states in the United States, 123 UTLAs in England, 26 states and 1 federal district in Brazil, and 32 countries worldwide to investigate the potential factors related to the mpox outbreaks. For historical analyses, data were obtained from approximately one decade ago (United States: 2012; England: 2014; Brazil: 2013; globe: 2013), with full details provided in *Supplementary Materials*.

### 2.3 Statistical analysis

To investigate the 2022 mpox outbreaks, we built multiple logistic regression models across four spatial levels—national (global), and subnational units in the United States, England, and Brazil. Given the absence of a universal outbreak definition, outbreak threshold values were set at 200, 10, 150, and 1000 for the United States, England, Brazil, and globe, respectively, aiming to balance case-control ratios while accounting for epidemiological variations across geographic scales. To mitigate multicollinearity, we employed standard variable selection procedures. To evaluate the robustness of our findings, we conducted sensitivity analyses using alternative thresholds and repeated logistic regression analyses using revised outbreak definitions and variable selection strategies. Additionally, we assessed the robustness of our results by performing multiple linear regression with mpox incidence as a continuous outcome. All analyses were performed using the stats package in R (version 4.2.1). Further details are provided in the *Supplementary Materials*.

## 3 Results

### 3.1 The association between multifaceted factors and the outbreak of mpox in 2022 for the United States, England, and Brazil

**Fig. 1:**
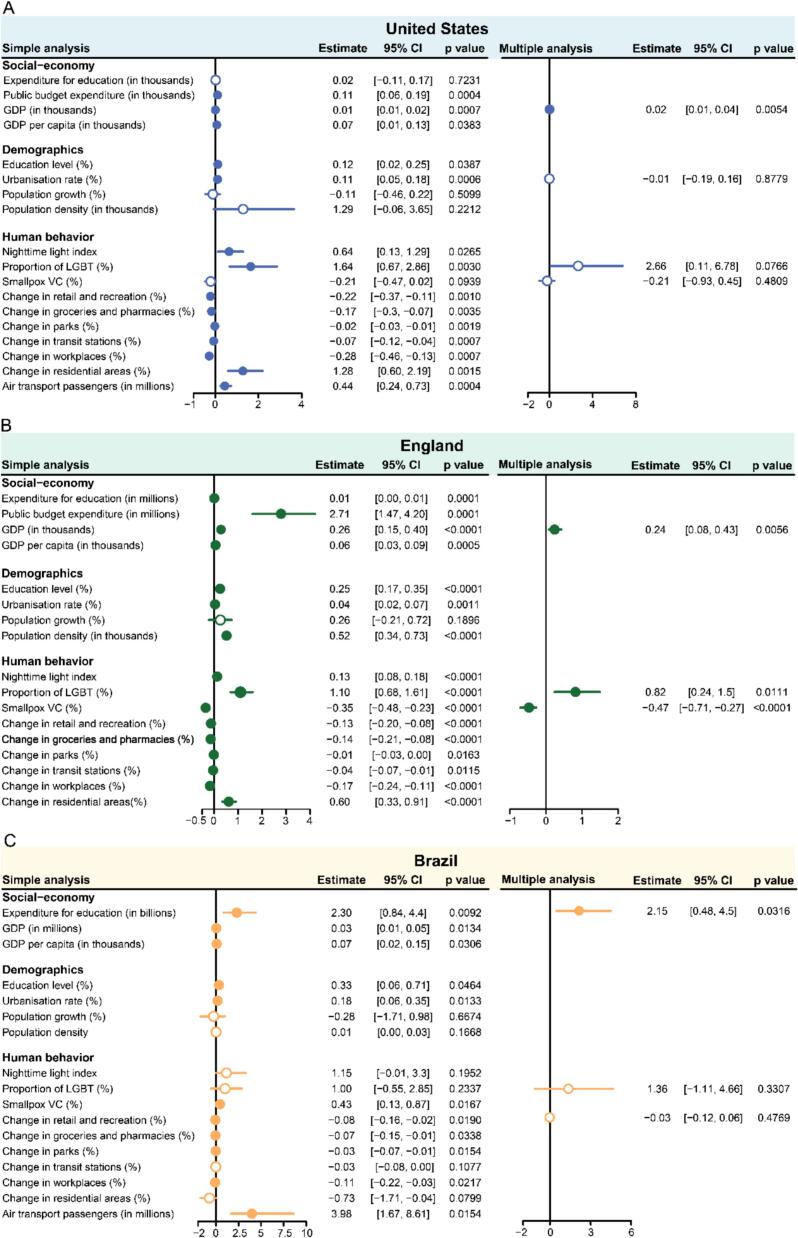
Simple and multiple analyses in the United States, England, and Brazil. (A) Simple and multiple logistic regression analysis at the state level in the United States. (B) Similar to (A), but at the UTLA level in England. (C) Similar to (A), but at the state level in Brazil. Solid circles represent significant (i.e., *P* < 0.05) values, while hollow circles represent insignificant values (i.e., *P* > 0.05). Bars show the 95 % CI. GDP: gross domestic product. Smallpox VC: smallpox vaccination coverage.

In 2022, mpox cases increased significantly compared to previous years, with cumulative case counts reaching over 29,000 in the United States, 3700 in the United Kingdom, and 10,000 in Brazil by December (Fig. S2). Notably, 94.0 %, 96.8 % and 84.1 % of mpox cases were reported among MSM in the United States, the United Kingdom and Brazil, respectively [[27], [28], [29]], suggesting that the outbreak dynamics in non-endemic regions may have been shaped by behavioral patterns [23]. To identify the specific factors associated with the probability of mpox outbreaks, we performed simple and multiple logistic regression analyses for the United States, England, and Brazil (Fig. 1). The analysis revealed a positive correlation (Pearson's correlation coefficient) between the probability of mpox outbreaks and the urbanization rate in the United States (Fig. S3, *R* *=* 0.43, *P* < 0.05), England (Fig. S3, *R* = 0.25, *P* < 0.05), and Brazil (Fig. S3, *R* = 0.52, *P* < 0.05). This finding is consistent with reports from Nigeria, where the 2017 resurgence of mpox was attributed to multiple factors, with rapid urbanization identified as a major driver of transmission [24]. Additionally, the probability of mpox outbreaks was also associated with the proportion of LGBT individuals in these countries (Fig. S3, the United States: *R* = 0.39, *P* < 0.05; England: *R* = 0.72, *P* < 0.05; Brazil: *R* = 0.26, *P* = 0.18). These findings suggested the role of the change of human behavior in 2022 mpox outbreak in non-endemic regions.

A significant negative correlation between the probability of mpox outbreaks and the smallpox vaccination coverage (estimated based on the actual smallpox vaccination cessation dates in real-world scenarios [30]) was observed in both the United States [Fig. 1, logistic regression coefficients: -0.21 (95 %CI:-0.47–0.02), *P* = 0.09] and England [Fig. 1, logistic regression coefficients: -0.35 (95 %CI:-0.48–0.23), *P* < 0.05], which was under expectation due to the cross-protection of smallpox vaccination against MPXV [31]. However, an interesting contrast was observed in the association with the smallpox vaccination coverage in Brazil, where it was positively associated with the probability of mpox outbreaks (Fig. 1). This difference may be partly attributed to the fact that in Brazil, a large proportion of the population, including most smallpox-vaccinated individuals (primarily those aged 60+), is concentrated in highly urbanized regions (Fig. S4) [32]. In addition, to address the uncertainty in the smallpox vaccination coverage estimation (similar to previous study [30]), two alternative scenarios were considered: one where all countries shared a routine smallpox vaccination cessation date of 1984 and another where all countries achieved 100 % vaccination coverage before cessation. Sensitivity analyses regarding the relationship between these two alternatives vaccine coverage estimations and the probability of mpox outbreaks were conducted and the conclusions remained consistent (Fig. S5). Furthermore, considering that the majority of the affected population of mpox was under 60 years old, we estimated the smallpox vaccination coverage for the age group 0–59 years and conducted the same analysis. The results showed that the smallpox vaccination coverage for the age group of 0–59 was also significantly associated with the probability of mpox outbreaks, consistent with the above analysis [Fig. S5, United States: logistic regression coefficients: -0.44 (95 %CI: −0.97-0.02), *P* = 0.06; England: logistic regression coefficients: -0.77 (95 %CI: −0.1.06–0.51), *P* < 0.0001; Brazil: logistic regression coefficients: 0.77 (95 %CI: 0.26–1.56), *P* < 0.05)].

We also observed a correlation between the nighttime light index (a proxy for human contact intensity [33]) and the probability of mpox outbreaks (Fig. 1). This suggested a plausible link between human behavior and the mpox outbreak. Notable associations were also showed in changes in mobility (reflecting human behavioral changes during outbreaks). Specifically, the probability of mpox outbreaks was negatively correlated with changes in mobility for transit stations, workplaces, retail and recreation areas, parks, and groceries and pharmacies areas. A possible explanation is that in economically developed regions with higher mpox transmission risk, mobility decreased more substantially during the outbreak period; however, this pattern may not be generalizable to all mpox-endemic countries [34]. However, a positive association was detected for residential areas (Fig. 1A and B), aligning with sexual contact being the primary transmission pattern in the 2022 mpox epidemic. Interestingly, the number of air transport passengers (representing long-distance connectivity that could facilitate mpox spread) was also positively related to the probability of mpox outbreaks in the United States and Brazil, consistent with previous studies [25]. In addition, the probability of mpox outbreaks was also positively related to economic and demographic factors, such as GDP and education level. These findings suggest that changes in various aspects of human behavior may be associated with the probability of mpox outbreaks.

Multiple logistic regression models were also built for the mpox outbreak in 2022. Considering the multicollinearity among the various factors, we selected four variables (GDP, urbanization rate, proportion of LGBT individuals and smallpox vaccination coverage) for the United States, three variables (GDP, proportion of LGBT individuals and the smallpox vaccination coverage) for England and three variables (expenditure for education, proportion of LGBT individuals and change in retail and recreation) for Brazil (see the *Supplementary Materials* for more details). These results, aligning with the above findings, highlighted the association of GDP, proportion of LGBT individuals, and smallpox vaccination coverage with the probability of mpox outbreak in the United States and England. To assess the robustness of our findings, we repeated logistic regression with an alternative outbreak definition and revised variable selection (Fig. S6), and additionally performed multiple linear regression treating mpox incidence rate as a continuous outcome variable (Fig. S7). The results were broadly consistent with the above analysis. In summary, these results implied that demographic and human behavior factors, such as higher urbanization rate, reduced smallpox vaccination coverage, and higher proportion of LGBT individuals, probably contributed to the 2022 mpox outbreak in non-endemic regions.

### 3.2 Comparing the probability of mpox outbreak between the historical years and 2022 for the United States, England, and Brazil

Based on the established multiple logistic regression model, the probability of mpox outbreaks was assessed for historical years and 2022 (see *Materials and Methods*). We note that mpox did not cause outbreaks in historical years; here, we focus on the probability of outbreak. In this analysis, we assumed that the biological characteristics of MPXV in historical years were same with those in 2022 and focused on the impact of human behavior on the mpox outbreak in a long-term way. Additionally, the performance of model was further evaluated through 3-fold cross-validation (see Fig. S8 A, B and C). The mean AUC values were 0.94 (United States), 0.91 (England), and 0.86 (Brazil).

**Fig. 2:**
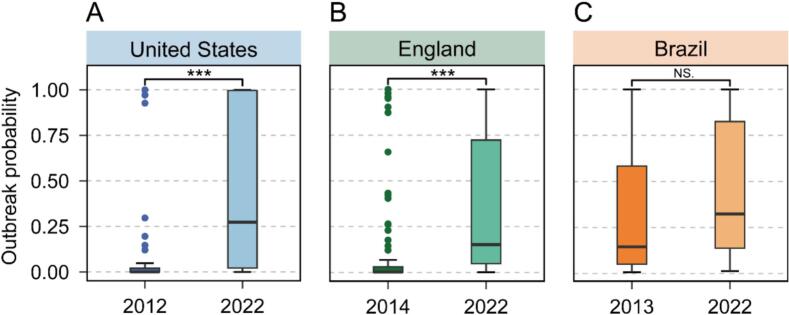
Comparison of the probability of mpox outbreak between historical years and 2022 in the United States, England, and Brazil. (A) Comparison of the probability of mpox outbreaks between 2012 and 2022 in the United States (an outbreak is defined if the number of mpox cases exceeds 200). (B) Comparison of the probability of mpox outbreaks between 2014 and 2022 in England (an outbreak is defined if the number of mpox cases exceeds 10). (C) Comparison of the probability of mpox outbreaks between 2013 and 2022 in Brazil (an outbreak is defined if the number of mpox cases exceeds 150). Each boxplot represents the distribution of outbreak probabilities. Each dot on the graph represents a state or a UTLA, *P* were calculated by the Wilcoxon rank-sum test. *** *P* < 0.001, NS, not significant. Note: sample size varies across analyses due to variable availability.

Our estimates suggested a significant increase in the probability of mpox outbreaks in the year of 2022 compared to historical years (Fig. 2). In the United States, the median probability of mpox outbreaks in 2022 was 0.27 (95 % CI: 0.04–0.95), increasing by approximately 913-fold compared to the probability observed in 2012 (Median: 0.0003, 95 % CI: 0.0001–0.0028) (Fig. 2A). Similarly, within England (Fig. 2B), a significant difference was observed between the median probability of mpox outbreaks in 2022 (Median: 0.15, 95 % CI: 0.10–0.28) and that in 2014 (Median: 0.0018, 95 % CI: 0.0010–0.0045), with an 83-fold increase in 2022. In Brazil, the median probability of mpox outbreak in 2022 (Median: 0.32, 95 % CI: 0.23–0.71) almost increased by 1-fold compared to the value of 0.15 (95 % CI: 0.06–0.47) observed in 2013 (Fig. 2C).

To account for the potential impact of different cutoff values used to define an outbreak, we performed sensitivity analyses using various cutoff values. These results consistently indicated a higher probability of a mpox outbreak in 2022 compared to historical years (Fig. S9). In addition, considering the impact of non-pharmaceutical interventions, sensitivity analysis was performed in the United States, revealing consistent patterns (Fig. S10A). These findings suggest that the shift in human behavior may contribute to an increased probability of mpox outbreaks.

### 3.3 Comparing the probability of mpox outbreaks between 2013 and 2022 at the country level

**Fig. 3:**
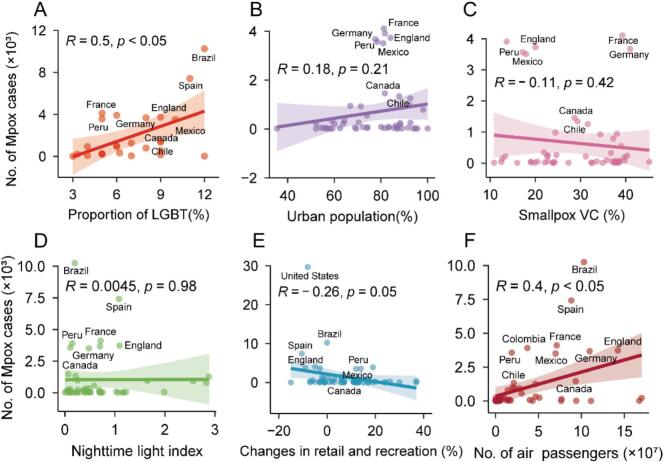
The association between the number of mpox cases and human behavior at the country level. (A) The association between the number of mpox cases and the proportion of LGBT individuals at the country level (*n* = 26). (B) The association between the number of mpox cases and the urbanization rate at the country level (*n* = 51). (C) The association between the number of mpox cases and the smallpox vaccination coverage at the country level (*n* = 53). (D) The association between the number of mpox cases and the nighttime light index at the country level (*n* = 50). (E) The association between the number of mpox cases and the changes in retail and recreation at the country level (*n* = 55). (F) The association between the number of mpox cases and the number of air passengers at the country level (n = 50). Each dot on the graph represents a country. The lines and gray-colored ribbons represent means and 95 % ranges, respectively. *R* represents the Pearson correlation coefficient, and *p* represents its significance level. VC = vaccination coverage. The smallpox vaccine coverage was estimated for all age groups. Note: sample size varies across analyses due to variable availability.

In the country-level analysis, both simple and multiple logistic regression analyses suggest that the probability of mpox outbreaks was positively associated with urbanization rate and the proportion of LGBT individuals, supporting the above conclusions (Fig. 3 and Fig. S11). Recognizing that the proportion of LGBT individuals may not fully reflect human sexual behavior at the population level, we also collected data on homosexuality acceptability and performed the same analysis. The number of mpox cases was still positively associated with homosexuality acceptability at the country level (Fig. S12), consistent with the previous conclusion. A similar negative trend between the probability of mpox outbreak and the smallpox vaccination coverage was also observed, despite non-significance (Fig. 3 and Fig. S11). We also repeated logistic regression with an alternative outbreak definition and revised variable selection, and additionally performed multiple linear regression treating mpox incidence rate as a continuous outcome variable. The results were aligned with the above analysis (Figs. S13, S14). These results extended previous conclusions to a global scale.

**Fig. 4:**
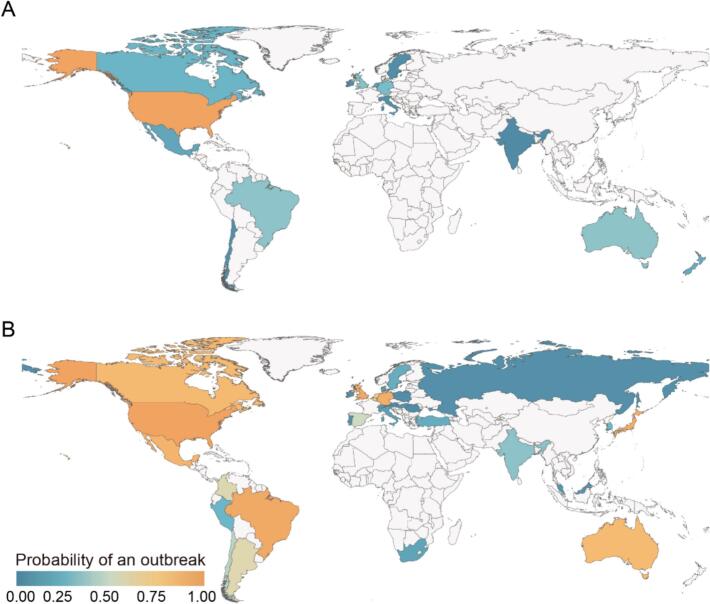
Comparison of the probability of mpox outbreaks between 2013 and 2022 globally. (A) The probability of a mpox outbreak in 2013 (*n* = 16) at the country level. (B) The probability of a mpox outbreak in 2022 (*n* = 32) at the country level. An outbreak is defined if the number of mpox cases exceeds 1000. Colors on the map indicate the estimated probability of outbreak in each country. Gray areas represent countries for which data were not available or were excluded from the analysis. Note: sample size varies across analyses due to variable availability.

Utilizing the constructed multiple logistic regression model (model evaluation using 3-fold cross-validation in Fig. S8D, the mean AUC values: 0.83), the probability of a mpox outbreak was also estimated for 2013 and 2022 (see *Materials and Methods*). Our estimates suggested the probability of mpox outbreaks in 2022 (Median: 0.28, 95 % CI: 0.14–0.58) surpassed that of 2013 (Median: 0.14, 95 % CI: 0.02–0.28)1-fold higher. For the year of 2013, due to lack of data, we estimated the probability of outbreaks for only 16 countries, with the United States being the only one to exceed 0.5 (Fig. 4A). In contrast, the probability of an outbreak for 2022 revealed substantial changes (Fig. 4B). In 2022, we estimated the probability of outbreaks for 32 countries, with 14 countries exceeding 0.5, and the United States reaching 1.00. Specifically, of the 14 countries assessed for the probability of an outbreak in both 2013 and 2022, 13 countries showed significantly higher outbreak probabilities in 2022. Considering the potential impact of different cutoff values used to define an outbreak, a sensitivity analysis was also conducted using different cutoff values at the country level. The results were consistent with the above analysis (Fig. S15). Additionally, the sensitivity analysis regarding the potential impact of control measures was also conducted globally, yielding consistent conclusion (Fig. S10B). These findings indicated that human behavior may have a critical impact on the probability of mpox outbreaks on the global scale in 2022.

## 4 Discussion

Despite a significant reduction of the current mpox cases, there remains a risk of a large resurgence of infection [9]. Therefore, it is important to understand the factors that contributed to the outbreak in 2022. In this study, we conducted a comprehensive, One Health–based analysis to evaluate the potential factors. Although our study primarily focuses on human behavioral, demographic, and socioeconomic factors, these are intrinsically linked to ecological and animal health dynamics within the broader One Health framework. Human activities reshape the interfaces between people, wildlife, and ecosystems, thereby influencing zoonotic transmission pathways. Using regression models, we investigated the association between multiple factors and the 2022 mpox outbreak while also estimating the probability of mpox outbreaks in historical years.

While mpox is not classified as a sexually transmitted disease (STD), it is noteworthy that sexual contact is a predominant transmission mechanism in the 2022 mpox outbreak [19], and there is evidence to support the potential pattern of sexual transmission in this outbreak [35]. Our study identified a positive association between the sexual behavior and the number of mpox cases at the population level. Furthermore, a cluster of clade I mpox virus infections associated with sexual contact was reported in the Democratic Republic of Congo in the year of 2023 [12]. Therefore, investigating the relevant factors associated with STDs may provide insights for a deeper understanding of the transmission dynamics of mpox.

Human behaviors drive the changes in the patterns of transmission of infectious diseases [36], leading to changes in the populations affected. For example, the susceptible population for AIDS has changed over time, from initially affecting white homosexual and bisexual men to injecting-drug users [36], and more recently, to GBMSM, who accounted for approximately 67 % of AIDS cases in 2021 [36]. Interestingly, changes in the affected population were also observed in mpox transmission. Historically in Africa, mpox primarily affected individuals living in forested areas (particularly near habitats of squirrels), household contacts of infected persons, male, an children under 15 years [1]. Probably influenced by changes in human behavior and the virus-host interactions [37], the primary affected population of mpox shifted to the GBMSM population in the 2022 outbreak [23]. However, it is essential to acknowledge that MPXV infections can occur in any population group through close contact, not exclusively limited to MSM populations [38].

The interactions of human, ecological and animal health may also affect the spread of infectious diseases [39]. Urbanization is one aspect of this interaction, as urban expansion can alter human-wildlife interfaces, thereby potentially increasing opportunities for zoonotic spillover of MPXV. In our study, we observed a positive correlation between the urbanization rate and the number of mpox cases, which aligned with previous research [24]. However, the underlying mechanisms driving this correlation may differ. Previous study suggested that rapid urbanization in endemic region led to changes in land use, increasing the frequency and extent of animal-human contact [24]. In contrast, the 2022 mpox outbreak primarily occurred in non-endemic countries [19]. This discrepancy may be attributed to the fact that cities with high urbanization rates are typically economically developed and culturally diverse, which may influence patterns of human mobility, social interactions, and ultimately the risk of zoonotic transmission. These factors could foster greater tolerance for GBMSM groups and a more complex sexual transmission network in such cities [40]. Given that sexual contact is the main route of transmission in 2022, this could explain the positive correlation between the increase in urbanization rate and the rise in mpox cases. Therefore, from a geographical perspective, the risk of mpox outbreaks may further increase in Asia and Africa, where rapid urbanization is underway [41].

Our findings suggest that changes in host in a long-term way may contribute to an increased probability of mpox outbreaks when compared to historical years. The upward trend of LGBT proportion and the waning immunity to smallpox may both contribute to the increased pool of susceptible individuals for MPXV. Therefore, it is important to enhance the vaccination coverage within the susceptible population, and thereby reducing overall susceptibility to the disease. An intriguing finding was found in Brazil, where smallpox vaccination coverage showed a positive association with mpox outbreak probability. This may reflect the differences in population distribution between developed and developing countries. In developed countries, the proportion of the population aged 60+ was negatively related to the urbanization rate, whereas in Brazil, most of the population (about 87.4 %) [32], including the predominantly smallpox-vaccinated elderly (aged 60+) [30], is concentrated in highly urbanized regions.

Our study has several limitations. First, we assumed data consistency across successive years in comparing mpox outbreak probabilities. Given the relative stability of key socioeconomic, demographic, and behavioral factors, this likely has limited impact on the conclusions. Second, historical outbreak probabilities were estimated assuming stable covariate-risk relationships over time and consistent epidemiological characteristics. While this assumption may introduce some uncertainty, it should be reasonable. However, it may to some extent limit the reproducibility and generalizability of the model. Third, varying cutoff values for defining outbreaks and differences in year-specific data may lead to incomparability in outbreak probabilities among the United States, England, Brazil, and globe. Nonetheless, the observed increase in outbreak probability in 2022 compared to historical years was consistent across all regions. Fourth, although definitions of some variables (e.g., LGBT proportion and air travel volume) may vary across countries, they are consistent within each country over time, supporting the reliability of our conclusions. Fifth, while country-level LGBT data were derived from different sources, we complemented them with homosexuality acceptability data to ensure robustness. Finally, besides human behavior, other factors such as political instability may also affect mpox transmission, but limited data currently prevent their inclusion in our model.

Our results carry important implications for public health policy and practice. The study suggests that increasing urbanization, higher proportions of LGBT populations, and low smallpox vaccination coverage played significant roles in the 2022 mpox outbreak. Within One Health framework, our modeling approach also provides a tool for assessing transmission risk for non-sexual routes, as factors such as vaccination coverage and urbanization, are relevant across all transmission pathways. However, the specific applicability of the model requires further validation. Importantly, our results highlight the necessity of long-term monitoring of human behavioral trends and population-environment interactions to improve the prediction and management of outbreaks in the context of interconnected human, animal and environmental health.

To conclude, our findings show that, across diverse geographical scales, urbanization, social networks, and low smallpox vaccination coverage not only affect disease transmission within human populations but may also interact with ecological and environmental factors of zoonotic spillover. Recognizing these interconnected factors is consistent with the One Health paradigm, highlighting that controlling mpox outbreaks requires coordinated efforts across human health, wildlife conservation, and ecosystem management. Understanding how human activities shape ecological interfaces and pathogen ecology is crucial for developing comprehensive prevention and control strategies.

## CRediT authorship contribution statement

**Yuxi Ge:** Writing – original draft, Visualization, Methodology, Formal analysis, Data curation. **Yifei Wang:** Validation, Formal analysis, Data curation. **Ziqin Zhou:** Visualization, Methodology, Data curation. **Zhirui Zhang:** Validation, Formal analysis. **Yunyu Tian:** Writing – review & editing, Methodology. **Yun Feng:** Writing – review & editing, Data curation. **Peiyi Wu:** Visualization, Validation, Data curation. **Yuxin Wang:** Visualization, Data curation. **Ziyan Liu:** Visualization, Data curation. **Bingying Li:** Visualization, Data curation. **Zengmiao Wang:** Writing – review & editing, Writing – original draft, Supervision, Funding acquisition, Conceptualization.

## Declaration of generative AI and AI-assisted technologies in the writing process

During the preparation of this work the author(s) used ChatGPT in order to improve readability and language. After using this tool/service, the authors reviewed and edited the content as needed and take full responsibility for the content of the published article.

## Funding

Funding for this study was provided by the National Science and Technology Major Project (2021ZD0111201); the 10.13039/501100001809National Natural Science Foundation of China (82204160); the 10.13039/501100012166National Key Research and Development Program of China (2022YFC2303803); the Research on Key Technologies of Plague Prevention and Control in Inner Mongolia Autonomous Region (2021ZD0006). The funders had no role in the study design, data collection and analysis, decision to publish, or preparation of the manuscript.

## Declaration of competing interest

None declared.

## Data Availability

The authors do not have permission to share data.

## Appendix A Supplementary data

The Supplementary Information includes Materials and Methods, Supplementary Analysis, Figures S1–S15, Tables S1–S6, and Supplementary References. Image 1
