# Supplementary material for: Associations between the 2022 global mpox outbreak and multifaceted factors: A multi-geographical retrospective study
Source: One Health. 2025 Sep 24;21:101224. doi: 10.1016/j.onehlt.2025.101224 (PMC12514515; doi:10.1016/j.onehlt.2025.101224)
Supplement: Supplementary file 1 — The Supplementary Information includes Materials and Methods, Supplementary Analysis, Figures S1–S15, Tables S1–S6, and Supplementary References. [file mmc1.docx]

**Supplementary appendix:**

**Associations between the 2022 global mpox outbreak and multifaceted factors: A multi-geographical retrospective study**

The file includes:

Materials and Methods

Supplementary Analysis

Figures S1 to S15

Tables S1 to S6

Supplementary References

**Table of Contents**

[Materials and Methods 3](#_Toc200808317)

[Study design 3](#_Toc200808318)

[Data collection 3](#_Toc200808319)

[Epidemiological data 3](#_Toc200808320)

[Socioeconomic and demographic data 3](#_Toc200808321)

[Sexual orientation data 4](#_Toc200808322)

[Smallpox vaccination coverage data 5](#_Toc200808323)

[Mobility data and nighttime light index data 5](#_Toc200808324)

[Statistical analyses for assessing the probability of mpox outbreaks 6](#_Toc200808325)

[Supplementary Analysis 8](#_Toc200808326)

[Sensitivity analysis 1: Association between the probability of mpox outbreaks and multifaceted factors under different outbreak definition 8](#_Toc200808327)

[Sensitivity analysis 2: Assessing the association between mpox incidence and multifactorial factors using multiple linear regression 8](#_Toc200808328)

[Sensitivity analysis 3: Estimation of outbreak probability based on different cutoff values 8](#_Toc200808329)

[Sensitivity analysis 4: Correlation between the number of mpox cases and the smallpox vaccination coverage under different scenarios 8](#_Toc200808330)

[Sensitivity analysis 5: The potential impact of control measures in the United States and globe 9](#_Toc200808331)

[Supplementary Figures 10](#_Toc200808332)

[Supplementary Tables 25](#_Toc200808333)

[Supplementary References 30](#_Toc200808334)

# Materials and Methods

## Study design

In this retrospective, observational study, we quantified the association between socioeconomic, demographic, and human behavior factors and the outbreak of mpox in 2022. We also compared the probability of a mpox outbreak between the year of 2022 and historical years at the state level in the United States, the upper tier local authority (UTLA) level in England, the state level in Brazil and the country level worldwide.

We conducted simple logistic regression models to investigate the association between the probability of mpox outbreaks and multifaceted factors, including socioeconomic factors (e.g., gross domestic product (GDP), public budget expenditure), demographic factors (e.g., urbanization rate), and human behavior factors (e.g., smallpox vaccination coverage, sexual behavior, nighttime light index, and mobility data) in the United States, England, Brazil, and the world, respectively.

For a comprehensive analysis, multiple logistic regression models were built for the mpox outbreak in 2022 for the United States, England, Brazil, and globe, to assess the impact of multifaceted factors on the probability of mpox outbreaks. To estimate the probability of mpox outbreaks in historical years, we replaced the regression variable data in the year of 2022 with the data in the year of 2012 for the United States, data in the year of 2014 for England, data in the year of 2013 for Brazil, and data in the year of 2013 for globe, respectively.

## Data collection

We collected the epidemiological data and various factors data potentially related to mpox outbreak for 50 states in the United States, 123 UTLAs in England, 26 states and 1 federal district in Brazil, and 32 countries worldwide to investigate the potential factors related to the mpox outbreaks.

### Epidemiological data

We collected the cumulative confirmed cases of mpox at the state level for the United States and Brazil, at the UTLA level for England, and at the country level worldwide. In the United States, we collected the cumulative confirmed cases of mpox between May 10, 2022 and December 28, 2022 (n=51, excluding non-US resident, Puerto Rico). In England, cumulative confirmed cases (May 7 to December 20, 2022) were collected from the Office for National Statistics (ONS) (Table S2). In Brazil, the cumulative confirmed cases data was collected for 26 states and 1 federal district until June 1st, 2023 from Emergency Operation Center (COE). At the country level, the cumulative confirmed cases data was collected until December 15, 2022 from USA Centers for Disease Control and Prevention. For the cumulative confirmed cases data at the country level, we excluded countries located in Africa and countries with fewer than 10 cumulative mpox cases and finally 56 countries were retained. Given the potential impact of non-pharmacological interventions on the mpox outbreak, we also collected the number of mpox cases in the United States and worldwide through the date when the WHO declared the mpox outbreak a Public Health Emergency of International Concern (PHEIC) on July 23, 2022, to conduct sensitivity analyses.

### Socioeconomic and demographic data

The socioeconomic and demographic data were collected for the United States, England, Brazil, and globe, including expenditure for education, public budget expenditure, GDP (gross domestic product), GDP (per capita), education level, urbanization rate, population growth, and population density (see Table S1, S2, S3 and S4 for more details). When analyzing the 2022 outbreak, data for the majority of variables were from the most recent year. However, since the urbanization data for 2022 were not available for England and Brazil, we estimated it with reasonable assumptions. For England, the urbanization rate in 2010 was used as a proxy for the urbanization rate in 2022. For Brazil, we estimated urbanization data in the year of 2021 based on the assumption that the trend in urbanization rate at the state level was consistent with that on the national scale, and that was shown as the following equation:

$${Urbanization}_{s, 2021}= {Urbanization}_{s,2010}\times{Urbanization}_{n,2021}/{Urbanization}_{n,2010}$$

where ${Urbanization}_{s, 2021}$ and ${Urbanization}_{s,2010}$ represent the state-level urbanization rate in 2021 and 2010, respectively. ${Urbanization}_{n,2021}$ and ${Urbanization}_{n,2010}$ represent the national-scale urbanization rate in 2021 and 2010, respectively (see Table S3 for more details). Additionally, due to the public budget expenditure data at the state level was not available in Brazil, we collected the remaining variables except it.

We also obtained data for the variables considered in the regression models for the United States (i.e., GDP, urbanization rate, proportion of LGBT individuals and smallpox vaccination coverage) in the year of 2012, for England (i.e., GDP, proportion of LGBT individuals and smallpox vaccination coverage) in the year of 2014, for Brazil (i.e., expenditure for education, proportion of LGBT individuals and change in retail and recreation) in the year of 2013, and for the world (i.e., GDP, urbanization rate, proportion of LGBT individuals, smallpox vaccination coverage, and change in retail and recreation) in the year of 2013, to estimate the outbreaks of mpox in historical years. Due to the data limitation, some variables had to be replaced with data from successive years.

### Sexual orientation data

Given the limitations of data availability, the proportion of LGBT individuals was used as a proxy for gay, bisexual and men who have sex with the men (GBMSM) data in our study. In the United States, the proportion of lesbian, gay, bisexual, and transgender (LGBT) individuals for the year of 2020 and 2012 were both collected at the state level from the Gallup Survey published by the Williams Institute (see Table S1 for details of data sources).

In England, we obtained data for the number of usual residents classified by sexual orientation, age, and gender at an upper tier local authority (UTLA) level from Census 2021 published by ONS. Through dividing the population who identified as lesbian, gay, bisexual, or other (LGB+) people by the total population, we calculated the proportion of LGB+ individuals per UTLA for the year of 2021. As the UTLA-level data on the proportion of LGB+ data was only available for the year 2021, we estimated UTLA-level proportion in the year of 2014 based on the region-level proportion of LGB+ individuals between 2014 and 2022, and the UTLA-level proportion of LGB+ individuals in the year of 2021, considering one assumption that the changing trend in the proportion of LGB+ individuals at the UTLA level aligns with that at the region level. Based on the assumption, we estimated the UTLA-level proportion of LGB+ individuals, and that is shown as the following equation:

${Proportion}_{u,2014}={Proportion}_{r,2014}\times{Proportion}_{u,2021}/{Proportion}_{r,2021}$,

where ${Proportion}_{u,2014}$ and ${Proportion}_{u,2021}$ represent a UTLA-level proportion of LGB+ individuals in 2014 and 2021, respectively. ${Proportion}_{r,2014}$ and ${Proportion}_{r,2021}$ represent the region-level proportion of LGB+ individuals in 2014 and 2021, respectively (see Table S2 for details of data sources).

At the international level, we initially obtained the proportion of LGBT individuals for 30 countries, 24 of them from the LGBT+ Pride 2021 Global Survey, 4 of them from the LGBT+ Pride 2023 Global Survey, the data for Austria from the ECRI report, and data for Denmark from a “Sex *i* Denmark” survey. For data in historical years, we initially collected data for 16 countries, including 14 countries from the OECD Social Indicators, the Netherlands and China from the Central Bureau of Statistics and the Blued Big Data White Paper, respectively.

To conduct a more comprehensive analysis, we supplemented the proportion of LGBT individuals for additional countries by estimation, including Brazil and India. For Brazil and India, we obtained the number of men who have sex with the men (MSM) population in historical years to represent the proportion of MSM (as a proxy for GBMSM data in our study). In summary, we obtained sexual orientation data for 32 countries in 2022 and for 18 countries in historical years. Additionally, recognizing that the proportion of LGBT individuals may not fully reflect the human sexual behavior on the population level globally, the homosexuality acceptability data in 2019 was obtained from the Pew Research Center (see Table S4 for details of data sources).

In Brazil, the proportion of people who identify their sexual orientation as homosexual or bisexual was obtained from the National Health Survey-2019 (see Table S3 for details of data sources). As the proportion of LGBT data in historical years were unavailable, we obtained the country-level proportion of MSM individuals in 2013 to estimate it, considering two assumptions: one that the country-level proportion of MSM individuals is approximately the same as the country-level proportion of LGBT individuals in 2013, and another that the changing trend in the proportion of LGBT individuals at the state level aligns with that at the country level. Based on these assumptions, we estimated the state-level proportion of LGBT individuals, and that is shown as the following equation:

${Proportion}_{s,2013}={Proportion}_{s,2019}\times{Proportion}_{c,2021}/{Proportion}_{c,2013}$,

where ${Proportion}_{s,2013}$ and ${Proportion}_{s,2019}$ represent a state-level proportion of LGB+ individuals in 2013 and 2019, respectively. ${Proportion}_{c,2013}$ and ${Proportion}_{c,2019}$ represent the country-level proportion of LGBT individuals in 2013 and 2019, respectively.

### Smallpox vaccination coverage data

Data on the smallpox vaccination coverage were obtained from a previous study[1]. The study generated a database of routine smallpox vaccination campaign cessation dates and estimates of smallpox vaccination coverage before cessation, and provided the estimated smallpox vaccination coverage in 2022 for each admin-1 level region in all countries. We obtained smallpox vaccination coverage in the year of 2022 for the United States, Brazil and worldwide. Based on this database and combined with age structure data for the year of 2010, we estimated the smallpox vaccination coverage data for the United States and worldwide in the year of 2010, and for England in both the year of 2010 and 2022.

### Mobility data and nighttime light index data

In our analysis, two types of mobility data were considered: the changes in mobility data and the number of air transport passengers (see Table S1, S2 and S3 for more details). The changes in mobility data were sourced from Google Mobility Reports from May 1, 2022 to October 15, 2022. The mobility database included changes in mobility across six different categories of places compared to the baseline (the baseline is the median value, for the corresponding day of the week, between 3 January, 2020 and 6 February, 2020), including retail and recreation, groceries and pharmacies, parks, transit stations, workplaces, and residential. The median value of the six types of changes in mobility from 1 May 2022 to 15 October 2022 was used to represent the mobility changes during this period in the United States, England, Brazil, and globe. The number of air transport passengers was also collected for the United States in the year of 2022, for Brazil in the year of 2019, and worldwide in the year of 2019 (lack of the data in 2022). For the United States, the passenger traffic of all airports in a state was collected as the number of air transport passengers at the state level. For Brazil, the total number of air tickets sold as a destination in a state was collected to represent the number of air transport passengers. At the international level, the number of air transport passengers includes both domestic and international aircraft passengers of air carriers registered in the country. Note that data on the number of air transport passenger in the United States and in the world include both inward and outward passengers, whereas in Brazil only inward passengers are included.

In addition, we also obtained data on the nighttime light index, reflecting the intensity of human contacts to a certain extent, as one of the indicators of human behavior for the United States, England, Brazil and worldwide in 2022.

## Statistical analyses for assessing the probability of mpox outbreaks

We used data in 2022 to fit multiple logistic regression via the Stats package in R (version 4.2.1) at the state-level for the United States, UTLA-level for England, state-level for Brazil and the country-level for globe. The generalized linear model with a logit link function is as follows:

$$\ln\left( \frac{P\left( Y_{i}=Cases>cutoff \right)}{1-P\left( Y_{i}=Cases>cutoff \right)} \right)=\alpha+\sum_{m} \beta_{m}{variable}_{mi}+\varepsilon_{i}$$

where $i$represents the state, UTLA or country *i* in the USA or Brazil, England, or globe, respectively. $m$ denotes the index of the variable in the regression model, $\beta_{m}$is the regression coefficients corresponding to the $m$-th variable. $\alpha$ is the intercept. ${variable}_{mi}$ represents the retained regression variables by selection. $\varepsilon_{i}$ denotes the error. $Cases$ represent the cumulative confirmed mpox cases. $P$ represents the probability of the cumulative confirmed mpox cases greater that the threshold of mpox outbreak. $cutoff$ is dichotomization threshold to define mpox outbreak. In the absence of a standardized definition for mpox outbreaks, and to balance the cases and controls in the logistic regression analysis while accounting for epidemiological variations across geographic scales, threshold values were set at 200, 10, and 150 (all corresponding to a standardized incidence rate of 0.4 cases per 10 000 population) for the United States, England, and Brazil, respectively. At the global level, in consideration of substantial heterogeneity within country and to reduce the risk of misclassification caused by localized clusters of cases, the threshold was elevated to 1 000 cases, corresponding to a standardized incidence rate of 0.96 cases per 10 000 population. To account for the potential impact of cutoff values used to define an outbreak, we performed sensitivity analyses by using various cutoff values.

We used the following procedures to select variables to avoid the multicollinearity in the multiple regression: firstly, we categorized candidate variables into three classes: social-economy, demographics, and human behavior. Given the complexity and diversity of human behavior, it was further divided into four types: sexual behavior (including proportion of LGBT individuals), immune protection (including smallpox vaccination coverage), mobility (including the changes in mobility and the number of air transport passengers), and the intensity of human contacts (including nighttime light index[2]). We then selected one or two variables with the highest correlations to the number of mpox cases from social-economy and demographics categories, as well as from each type of human behavior; secondly, we calculated pairwise correlations among the retained variables; thirdly, we retained the variable with a higher correlation with the number of mpox cases in cases where the correlation between two variables exceeded 0.5; fourthly, we calculated the variance inflation factor (VIF) for all retained variables and deleted the variables with a VIF score of greater than or equal to 5. After this procedure, we selected four variables (GDP, urbanization rate, proportion of LGBT individuals, and smallpox vaccination coverage) for the United States, three variables (GDP, proportion of LGBT individuals, and smallpox vaccination coverage) for England, three variables (expenditure for education, proportion of LGBT individuals and change in retail and recreation) for Brazil, and five variables (GDP, urbanization rate, proportion of LGBT individuals, smallpox vaccination coverage, and change in retail and recreation) for the global analysis (Figure 2 and 3).

Furthermore, utilizing the constructed multiple logistic regression models, the historical year data were used to estimate the probability of mpox outbreaks in those historical years in R (version 4.2.1). We estimated the probability of mpox outbreaks for the United States in the year of 2012, England in the year of 2014, Brazil in the year of 2013, and globe in the year of 2013. These years were selected as they reflect conditions about a decade ago, enabling analysis of long-term trends and outbreak risks, and represent the most recent periods with adequate, reliable data for each geographical scale. Considering the impact of model stability on the accuracy of outbreak probability estimation, the performance of models was evaluated using k-fold cross-validation (Figure S8).

In addition, we repeated the logistic regression analysis using the median incidence (0.28) across four geographic scales as an alternative threshold for defining outbreaks. The candidate variables were categorized followed the main analysis. To avoid the multicollinearity, we applied least absolute shrinkage and selection operator (LASSO) regression for variable selection. The variables identified via LASSO were subsequently included in a multiple logistic regression model. Multicollinearity among variables was assessed using variance inflation factors. In cases of high collinearity, variables were retained based on prior knowledge and the goodness of model fit, as evaluated using the Akaike Information Criterion (AIC).

To assess the robustness of our findings, we performed multiple linear regression treating mpox incidence rate as a continuous outcome variable. The regression model was constructed through the following procedure. First, all candidate variables were standardized prior to analysis, and Pearson correlation coefficients were calculated to preliminarily assess linear relationships between the various factors and incidence. Second, candidate variables were categorized in the same way as in the main analysis, and then selected using LASSO regression. Finally, the selected variables were included in the multiple linear regression model, followed by a comprehensive set of diagnostic procedures, including outlier detection, multicollinearity assessment, residual analysis, normality testing, and heteroscedasticity testing. In the presence of multicollinearity, redundant variables were removed based on prior knowledge. If the normality assumption was violated, we re-estimated the model using a bootstrap regression approach to ensure the robustness of inference.

# Supplementary Analysis

## Sensitivity analysis 1: Association between the probability of mpox outbreaks and multifaceted factors under different outbreak definition

In addition to different numerical cutoffs as outbreak threshold in main analysis, we also explored an alternative approach by using the median incidence rate (median value: 0.28 cases per 10,000 population) at all geographic scale as the outbreak threshold. This method accounts for variations in population size and disease burden across regions. The results under this scenario were consistent with those of the main analysis, further supporting our conclusion that demographic and human behavior factors contributed to the 2022 mpox outbreak in non-endemic regions (Figure S6). Collectively, these findings indicate that our conclusions are robust to the choice of outbreak definition.

## Sensitivity analysis 2: Assessing the association between mpox incidence and multifactorial factors using multiple linear regression

To assess the robustness of our primary findings, we conducted a sensitivity analysis employing multiple linear regression to examine the association between mpox incidence and a range of potential explanatory variables (Figure S7). The results revealed broadly consistent patterns of association, thereby reinforcing the robustness of our conclusions across different model specifications and statistical frameworks.

## Sensitivity analysis 3: Estimation of outbreak probability based on different cutoff values

In the main analysis, we discussed the probability of outbreaks in the United States (cutoff = 200), England (cutoff = 10), Brazil (cutoff=150) and the world (cutoff = 1000) respectively. Here, we assessed the sensitivity of the probability of mpox outbreaks at different cutoff values used to define an outbreak (Figure S9 and S15).

In the United States, we used cutoff values of 100, 300, and 500, respectively. Our results showed that the probability of outbreaks in 2022 were consistently higher than that in 2012. In England, the results with cutoff values of 5, 20 and 30 were almost identical to the main analysis. Similarly, with cutoff values of 200, 300 and 500, the probability of outbreaks in 2022 for Brazil surpass that in 2013, despite no-significance. At the country level, the probability of an outbreak in 2022 has increased compared to 2013, regardless of whether the cutoff value was set at 300, 500 or 2000. These results suggest that using different cutoff values for mpox outbreaks doesn’t alter our conclusions.

## Sensitivity analysis 4: Correlation between the number of mpox cases and the smallpox vaccination coverage under different scenarios

In the main analysis, the smallpox vaccination coverage was estimated based on the real-world smallpox vaccination cessation dates[1]. To address the uncertainty in the smallpox vaccination coverage estimate, we additionally estimated the smallpox vaccination coverage in the United States, England, Brazil, and the world, under two alternative scenarios: one where all countries shared a routine smallpox vaccination cessation date of 1984 and another where all countries achieved 100% vaccination coverage before cessation (Figure S5).

In the United States, the results showed a negative association between the smallpox vaccination coverage and the number of mpox cases, which aligned with our main analysis. Almost identical to the main analysis, a significant negative association between the smallpox vaccination coverage and the number of mpox cases was detected in England. Similar to the main analysis, a positive trend between mpox cases and the smallpox vaccination coverage was also observed in Brazil. At the country level, consistent with the main analysis, the number of mpox cases was negatively associated with the smallpox vaccination coverage, although it did not reach statistical significance. The results suggested that changing the assumptions of vaccination estimate does not lead to a significant change in conclusions.

In addition, since the smallpox vaccination coverage was primarily contributed to people aged 60 over, we also estimated the smallpox vaccination coverage for the age group of 0-59 to assess the relationship between it and the number of mpox for the United States, England, and Brazil. The results aligned with our main analysis (Figure S5).

## Sensitivity analysis 5: The potential impact of control measures in the United States and globe

In the main analysis, we conducted the multiple regression analysis and evaluate the probability of mpox outbreak both in 2022 and the historical years. Given the potential impact of non-pharmacological interventions on the mpox outbreak, we obtained the number of mpox cases in the United States and globe up to the date when the WHO declared the mpox outbreak a PHEIC on July 23, 2022. We assumed that the number of mpox cases wouldn’t be affected by any control measures before July 23, 2022.

Similar to the main analysis, we conducted multiple logistic regression based on mpox case data up to July 23, 2022, estimating the probability of mpox outbreaks in both 2022 and historical years for the United States and globally. The pattern consistent with the main analysis was observed (Figure S10). These results indicated that human behavior plays an important role on the increasing probability of mpox outbreaks in 2022.

# Supplementary Figures


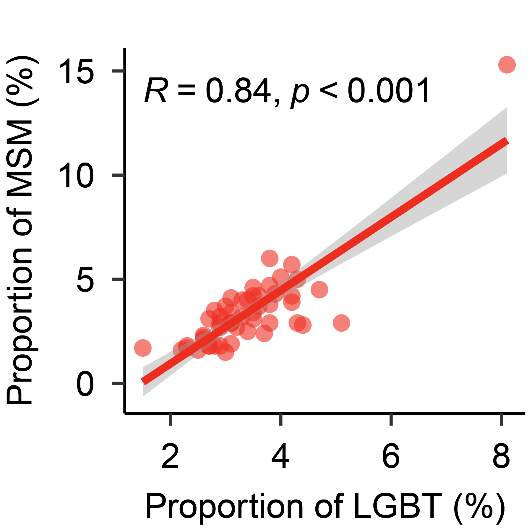


**Supplementary figure 1. The association between the proportion of LGBT individuals and the proportion of MSM individuals at the state level (n=51).** R represents the Pearson correlation coefficient, and p represents its significance level. Each dot on the graph represents a state.

**
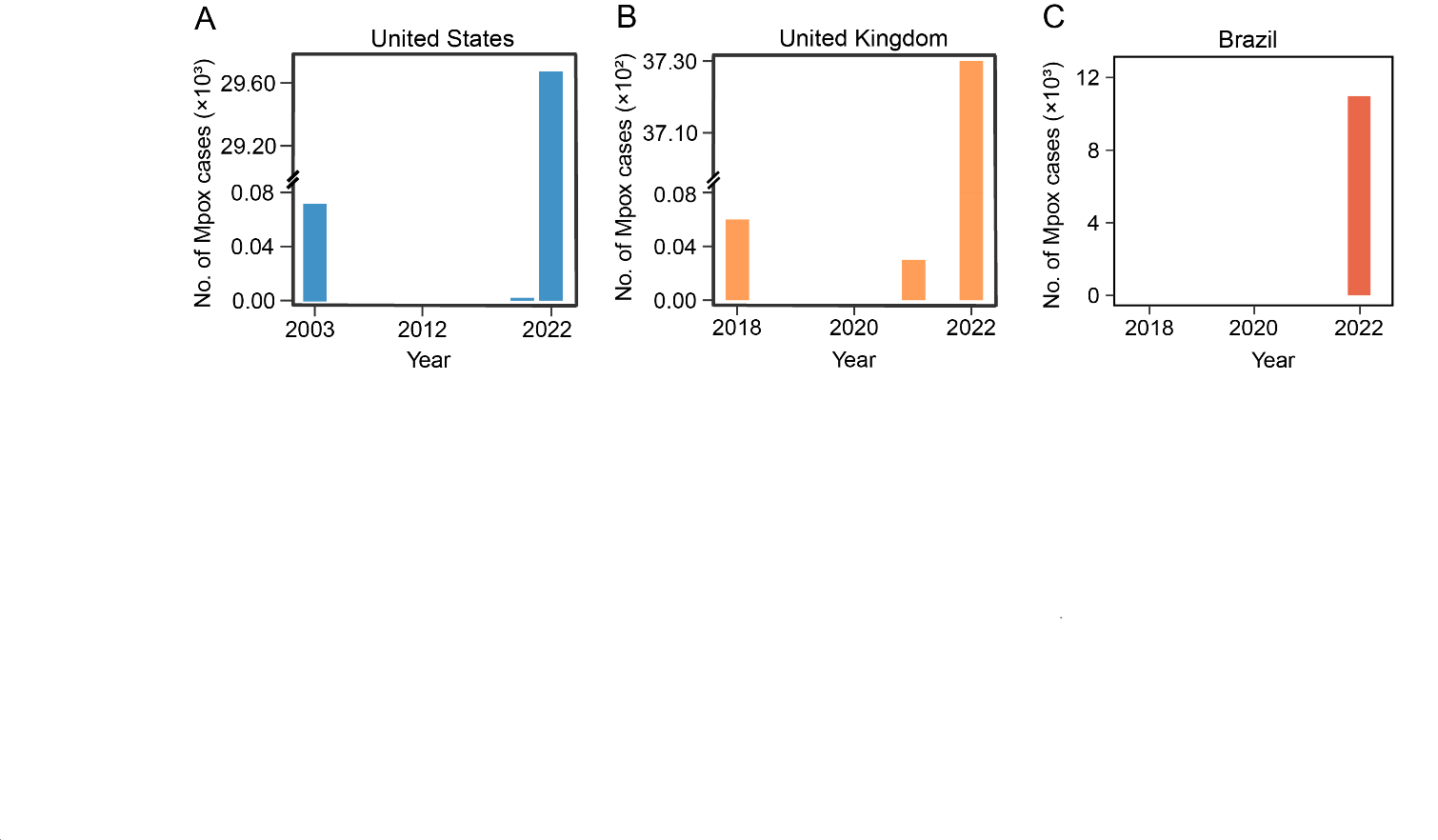
**

**Supplementary figure 2. Temporal distribution of MPXV infections in the United States, the United Kingdom and Brazil over the past years.** (**A**) Transmission and circulation of mpox since 2003 in the United States. (**B**) Transmission and circulation of mpox since 2018 in the United Kingdom. (**C**) Transmission and circulation of mpox since 2018 in Brazil. The bars represent the number of mpox cases.

***
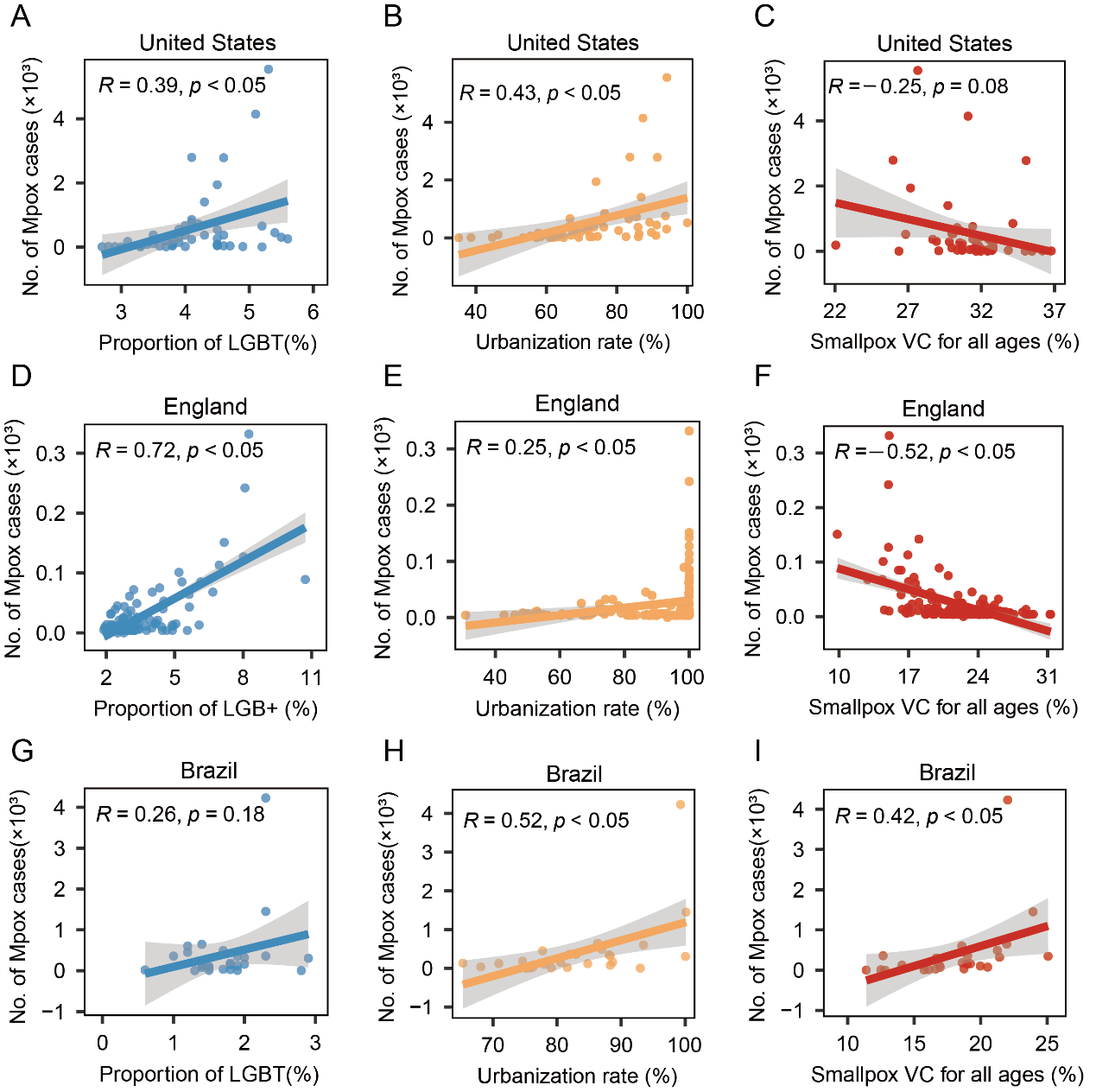
***

**Supplementary figure 3**. **The association between the number of mpox cases and human behavior**. (**A**) The association between the number of mpox cases and LGBT proportion at the state level in the United States (n=50). (**B**) The association between the number of mpox cases and the urbanization rate at the state level in the United States (n=51). (**C**) The association between the number of mpox cases and the smallpox vaccination coverage at the state level in the United States (n=51). (**D**) Similar to (A), but at the UTLA level in England (n=147). (**E**) Similar to (B), but at the UTLA level in England (n=145). (**F**) Similar to (C), but at the UTLA level in England (n=145). (**G**) Similar to (A), but at the state level in Brazil (n=27). (**H**) Similar to (B), but at the state level in Brazil (n=27). (**I**) Similar to (C), but at the state level in Brazil (n=27). Each dot on the graph represents a state, district, state, either in the United States, England, Brazil, respectively. The lines and grey-colored ribbons represent means and 95% ranges, respectively. R represents the Pearson correlation coefficient, and p represents its significance level. VC: vaccination coverage.


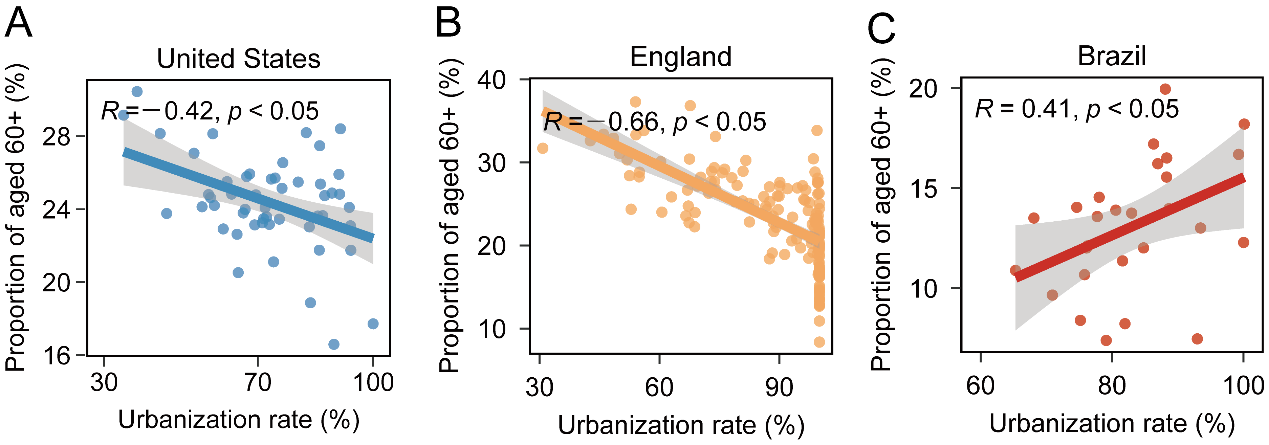


**Supplementary figure 4. The association between the urbanization rate and the proportion of people aged over 60.** (**A**) The association between the urbanization rate and the proportion of people aged over 60 in the United States (n=51). Each dot on the graph represents a state. (**B**) The association between the urbanization rate and the proportion of people aged over 60 in England (n=145). Each dot on the graph represents a UTLA. (**C**) The association between the urbanization rate and the proportion of people aged over 60 in Brazil (n=27). Each dot on the graph represents a state. R represents Pearson correlation coefficient, and p represents its significance level.


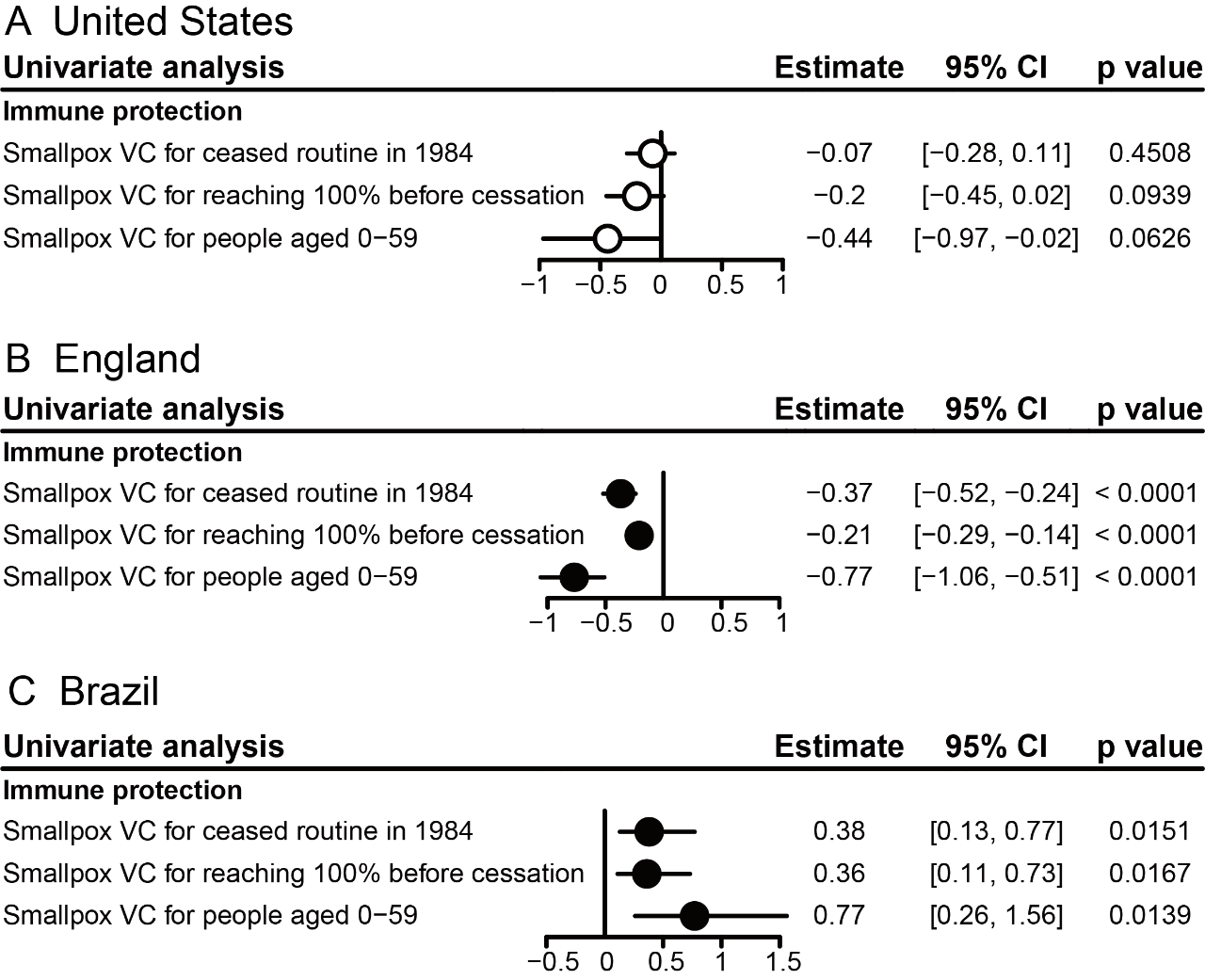


**Supplementary figure 5. The association between the probability of mpox outbreaks and the smallpox vaccination coverage estimated under three scenarios.** (**A**) The association between the probability of mpox outbreaks and the smallpox vaccination coverage estimated under three scenarios in the United States. (**B**) The association between the probability of mpox outbreaks and the smallpox vaccination coverage estimated under three scenarios in England. (**C**) The association between the probability of mpox outbreaks and the smallpox vaccination coverage estimated under three scenarios in Brazil. Solid circles represent significant (i.e., P<0.05) values, while hollow circles represent insignificant values (i.e., P>0.05). Bars show the 95% CI. Smallpox VC: smallpox vaccination coverage. Note that both the smallpox vaccination coverage for ceased routine in 1984 and for reaching 100% before cessation were estimated for all age groups.


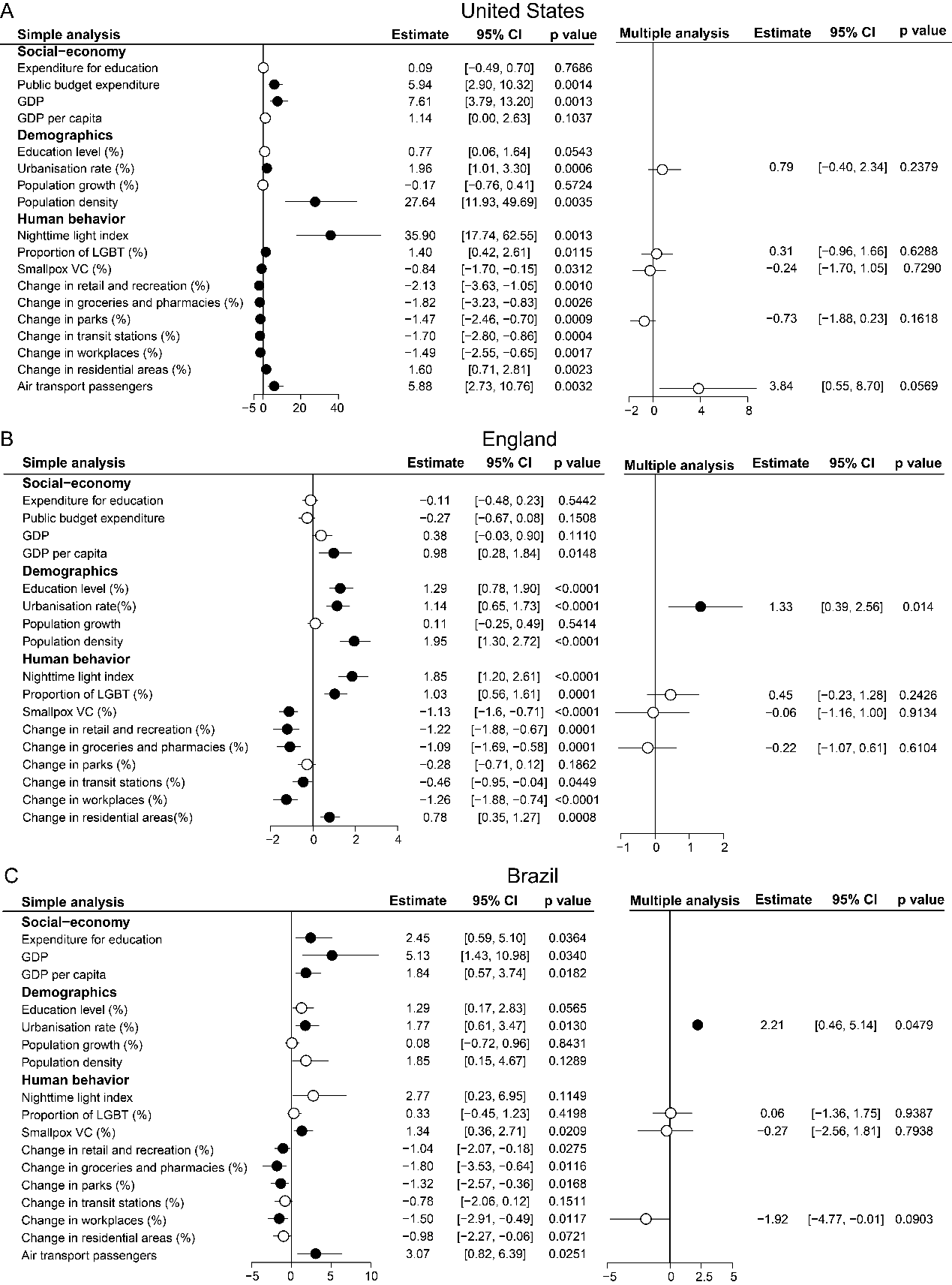


**Supplementary figure 6. Simple and multiple analyses in the United States, England, and Brazil.** (A) Simple and multiple logistic regression analysis at the state level in the United States. (B) Similar to (A), but at the UTLA level in England. (C) Similar to (A), but at the state level in Brazil. Solid circles represent significant (i.e., P<0.05) values, while hollow circles represent insignificant values (i.e., P>0.05). Bars show the 95% CI. GDP: gross domestic product. Smallpox VC: smallpox vaccination coverage. Note: the thresholds of outbreak were defined as the median incidence rate (0.28 cases per 10,000 population).

**
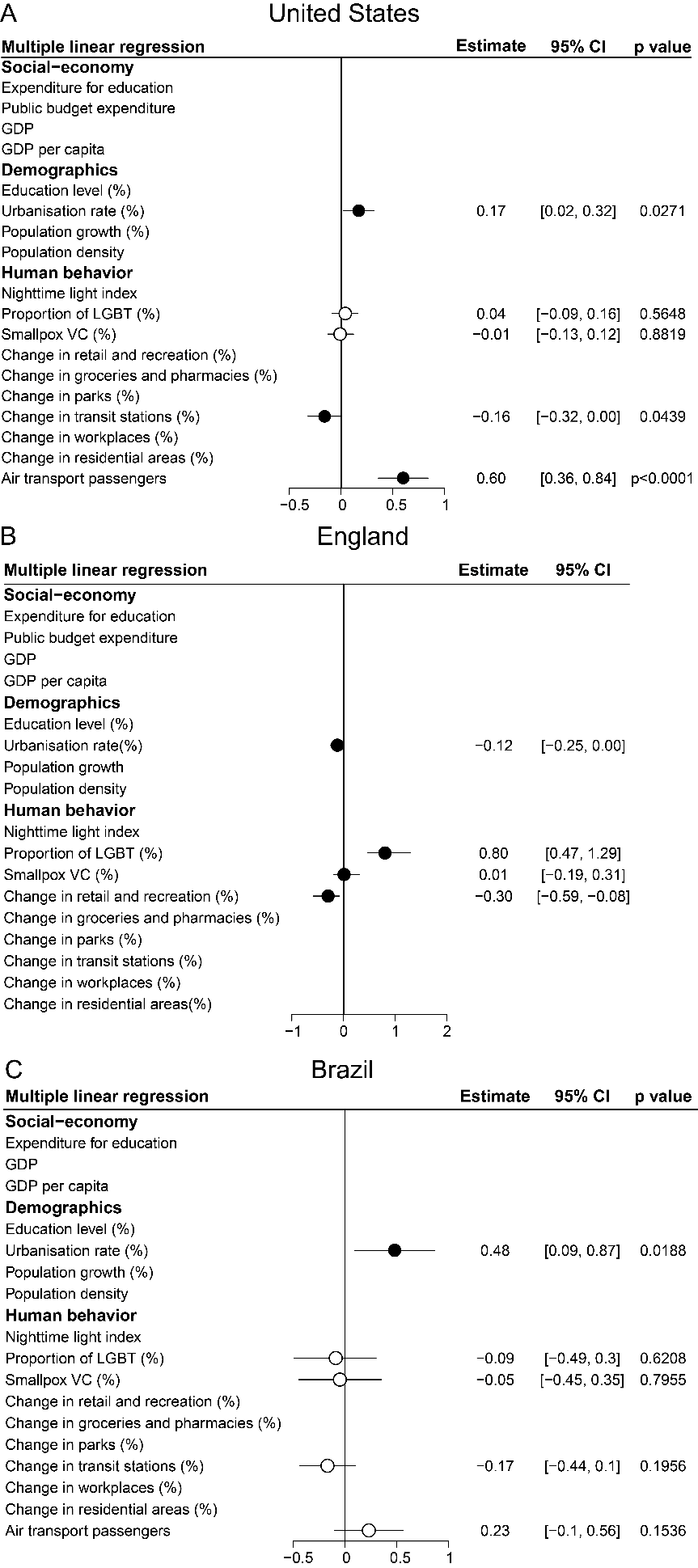
**

**Supplementary figure 7. Multiple linear regression analyses in the United States, England, and Brazil.** (A) Multiple linear regression analysis at the state level in the United States. (B) Similar to (A), but at the UTLA level in England. Bootstrap regression was used in (B), so P are not reported. (C) Similar to (A), but at the state level in Brazil. Solid circles represent significant (i.e., P<0.05) values, while hollow circles represent insignificant values (i.e., P>0.05). Bars show the 95% CI. GDP: gross domestic product. Smallpox VC: smallpox vaccination coverage.


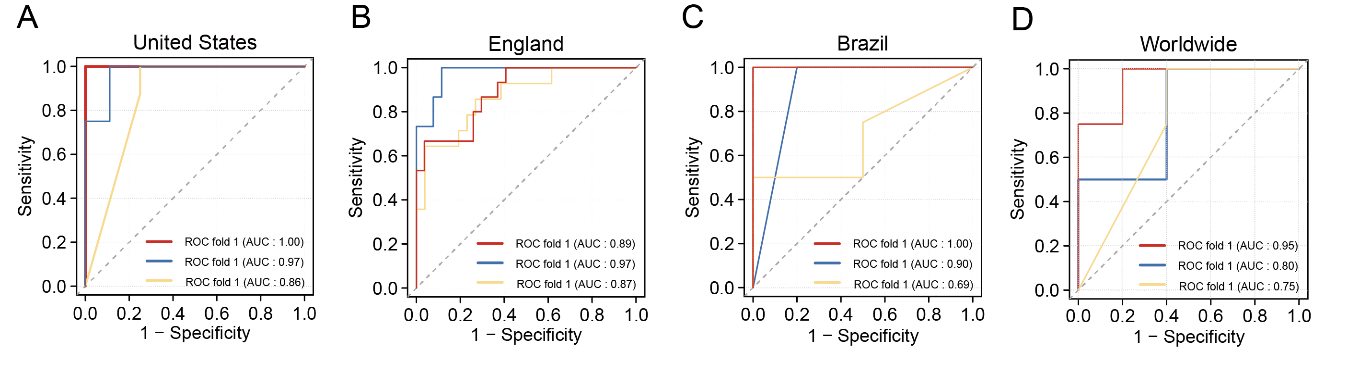


**Supplementary figure 8. The cross-validation for multiple regression models in the United States, England, Brazil and worldwide. (A)** The 3-fold cross-validation for multiple regression models at the state level in the United States. (**B**) Similar to (A), but at the UTLA level in England. (**C**) Similar to (A), but at the state level in Brazil. **(D)** Similar to (A), but at the country level. Note that due to the small sample size, the ROC curve for the United States, Brazil and worldwide are slightly different from that for England.


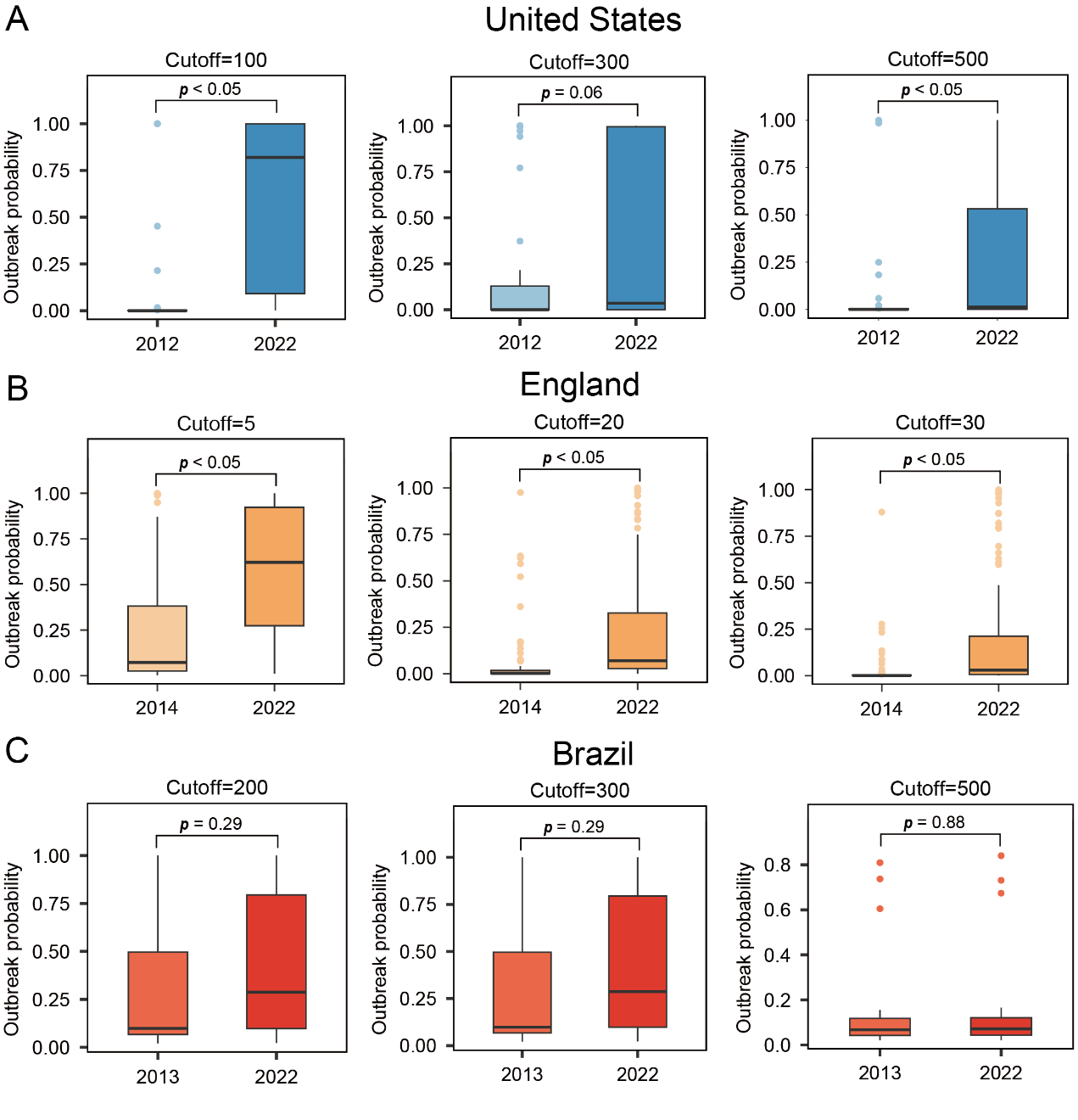


**Supplementary figure 9. Comparison of the probability of mpox outbreaks between the historical years and 2022 under different cutoff values.** (**A**) Comparison of the probability of mpox outbreaks between 2012 (n=51) and 2022 (n=50) in the United States. Each dot on the graph represents a state, p values were calculated by the Wilcoxon rank-sum test. (**B**) Comparison of the probability of mpox outbreaks between 2014 (n=116) and 2022 in England (n=123). Each dot on the graph represents a UTLA, p values were calculated by the Wilcoxon rank-sum test. (**C**) Comparison of the probability of mpox outbreaks between 2013 (n=27) and 2022 (n=27) at the state level in Brazil. Each dot on the graph represents a state, p values were calculated by the Wilcoxon rank-sum test. We assume that the proportion of LGBT individuals in each country is similar in the successive year in this analysis.

**
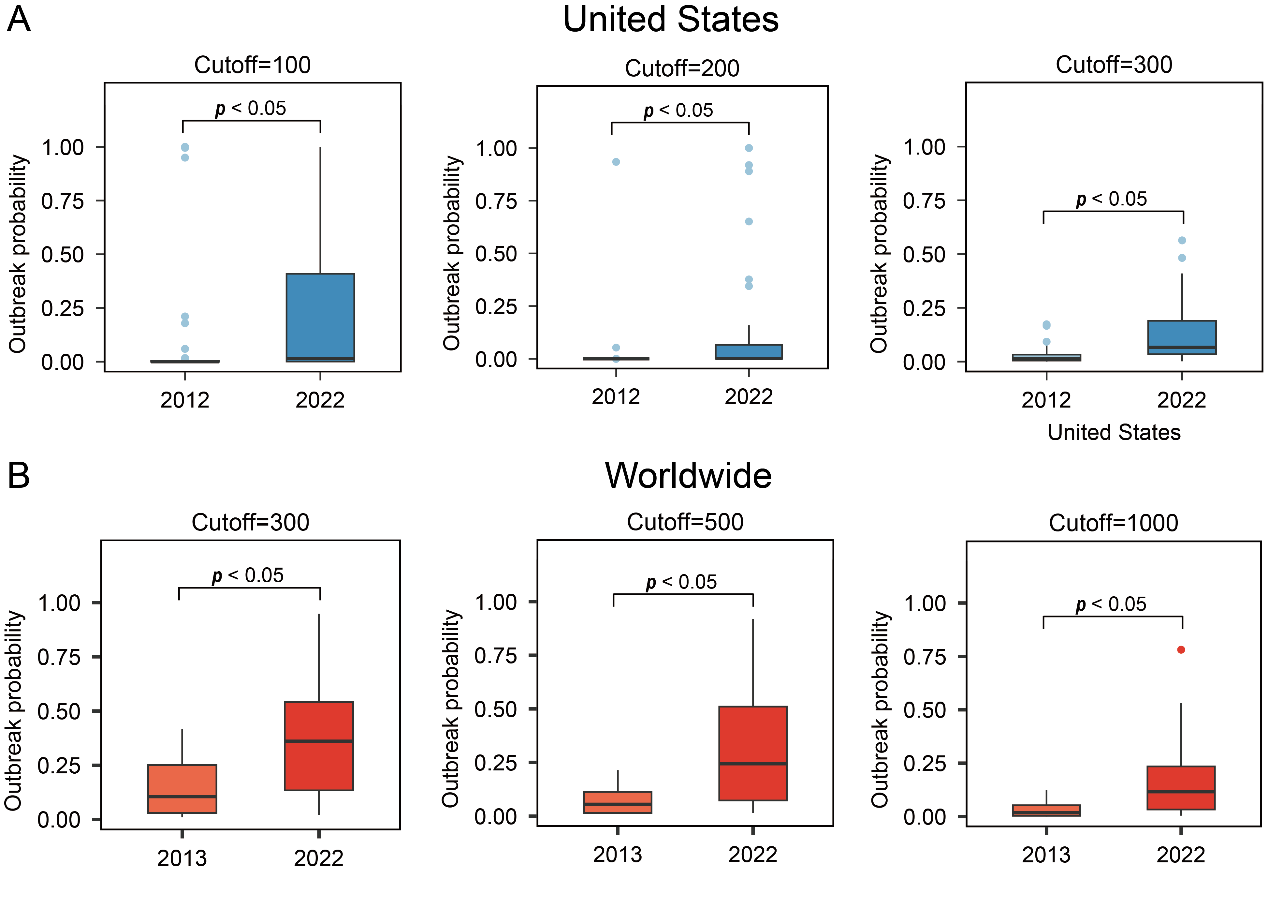
**

**Supplementary figure 10. Comparison of the probability of mpox outbreaks between historical years and 2022 based on the number of mpox cases until July 23, 2022.** (**A**) Comparison of the probability of mpox outbreaks between 2012 (n=48) and 2022 (n=51) at the state level in the United States. Each dot on the graph represents a state, P were calculated by the Wilcoxon rank-sum test. (**B**) Comparison of the probability of mpox outbreaks between 2013 (n=16) and 2022 (n=32) at the country level. Each dot on the graph represents a country, P were calculated by the Wilcoxon rank-sum test.


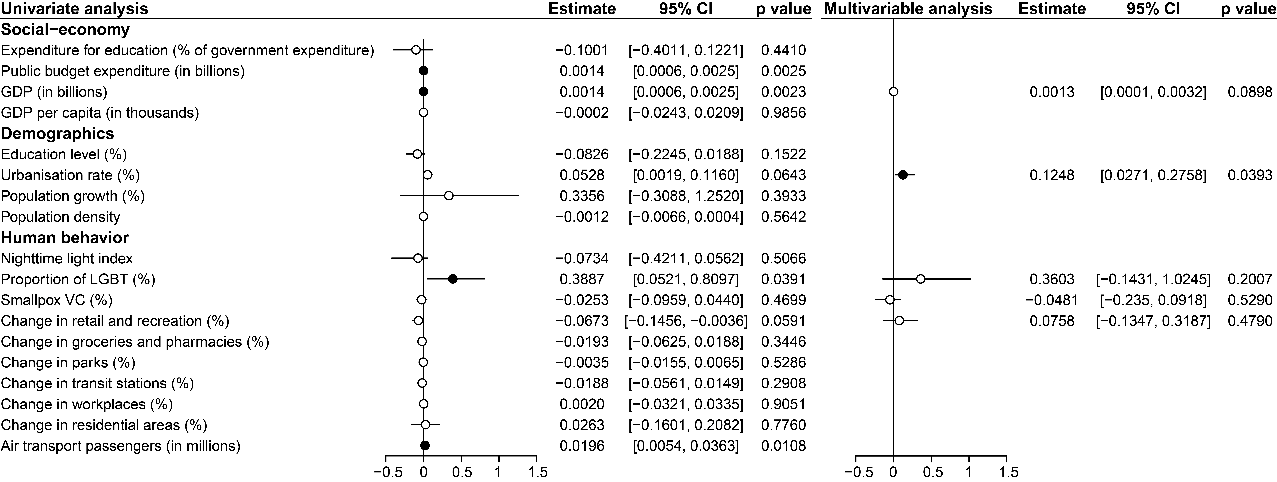


**Supplementary figure 11. Simple and multiple analyses for the probability of mpox outbreaks at the country level**. Solid circles represent significant (i.e., P<0.05) values, while hollow circles represent insignificant values (i.e., P>0.05). Bars show the 95% CI. GDP: gross domestic product. VC=vaccination coverage. The smallpox vaccine coverage was estimated for all age groups.


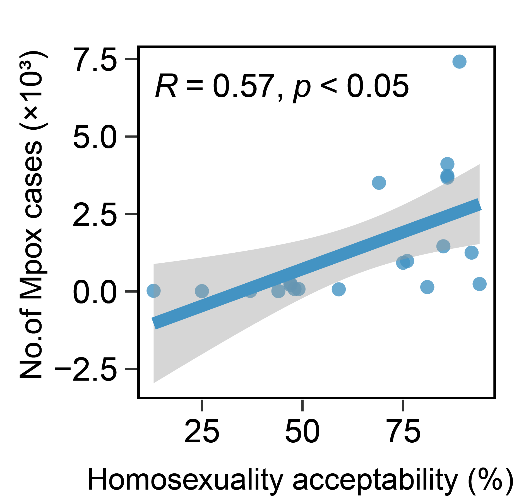


**Supplementary figure 12. The association between the number of mpox cases and homosexuality acceptability at the country level (n=20).** R represents the Pearson correlation coefficient, and P represents its significance level. Each dot on the graph represents a country.

*
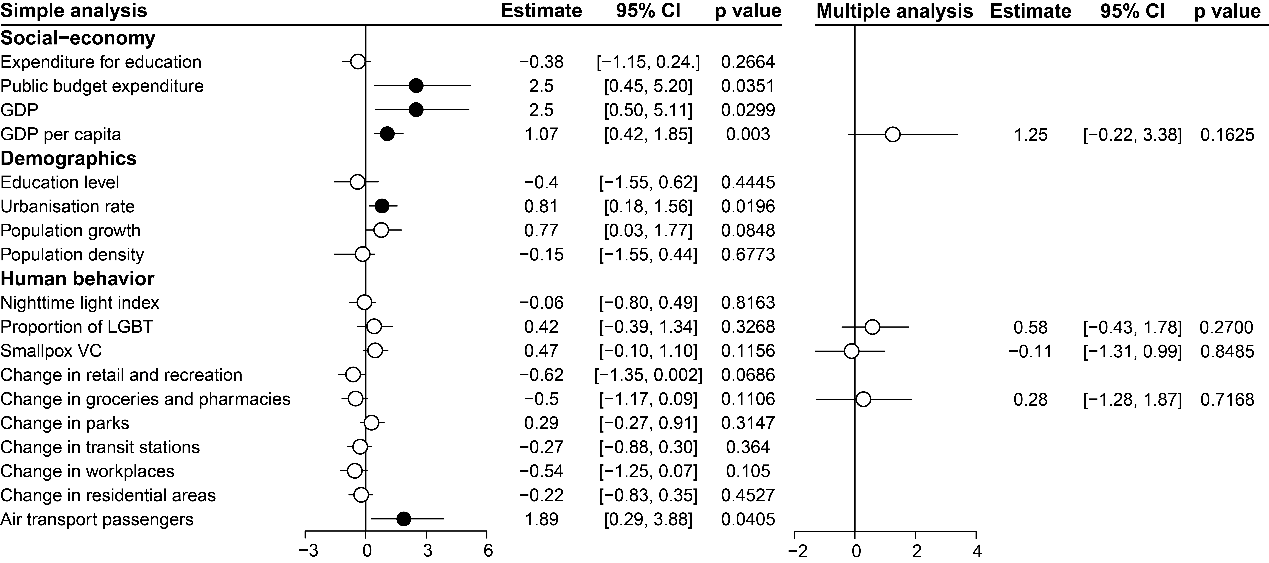
*

**Supplementary figure 13. Simple and multiple analyses for the probability of mpox outbreaks at the country level.** Solid circles represent significant (i.e., P<0.05) values, while hollow circles represent insignificant values (i.e., P>0.05). Bars show the 95% CI. GDP: gross domestic product. VC=vaccination coverage. The smallpox vaccine coverage was estimated for all age groups. Note: the threshold of outbreak was defined as the median incidence rate (0.28 cases per 10,000 population).


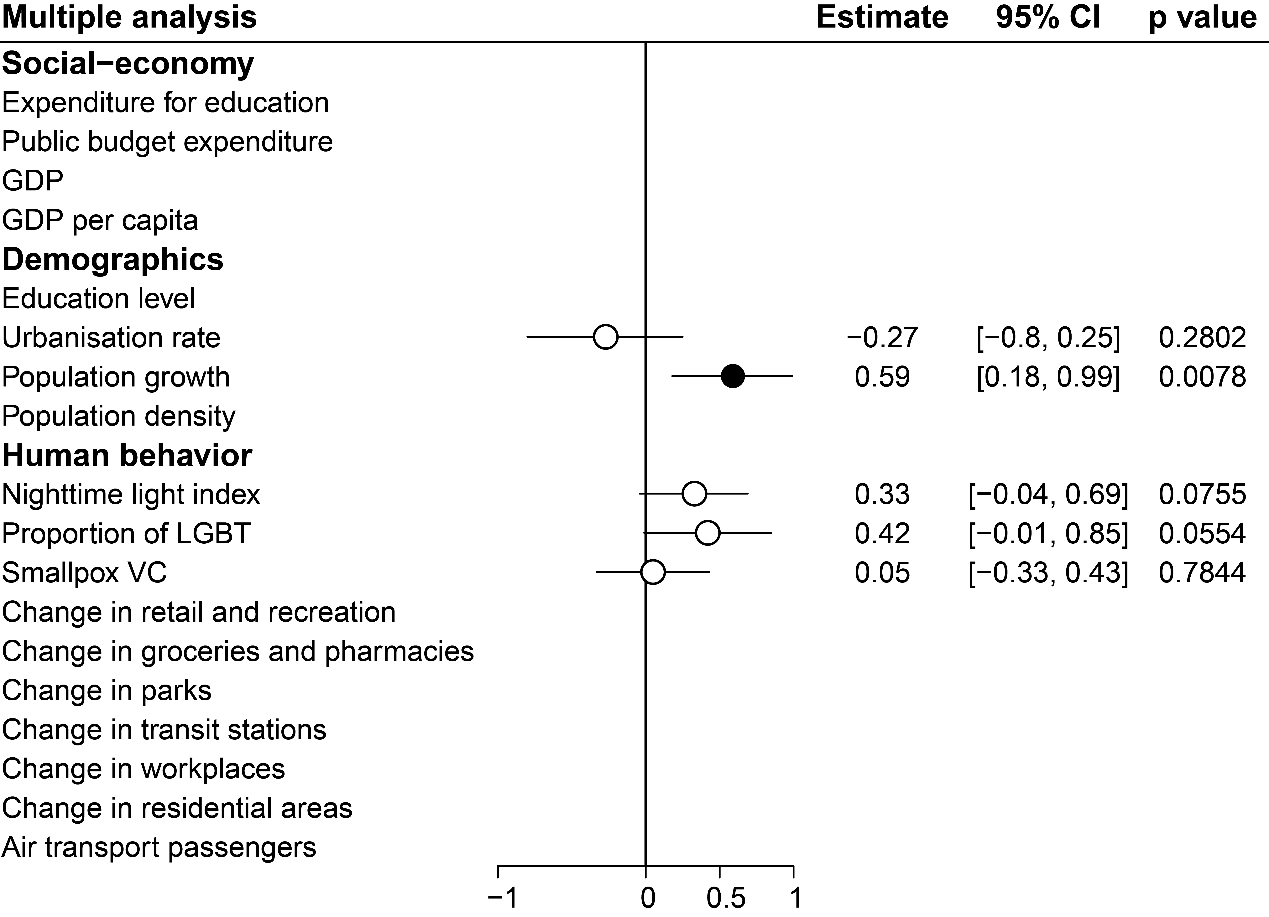


**Supplementary figure 14. Multiple linear regression analyses for the number of mpox cases at the country level.** Solid circles represent significant (i.e., P<0.05) values, while hollow circles represent insignificant values (i.e., P>0.05). Bars show the 95% CI. GDP: gross domestic product. VC=vaccination coverage. The smallpox vaccine coverage was estimated for all age groups.


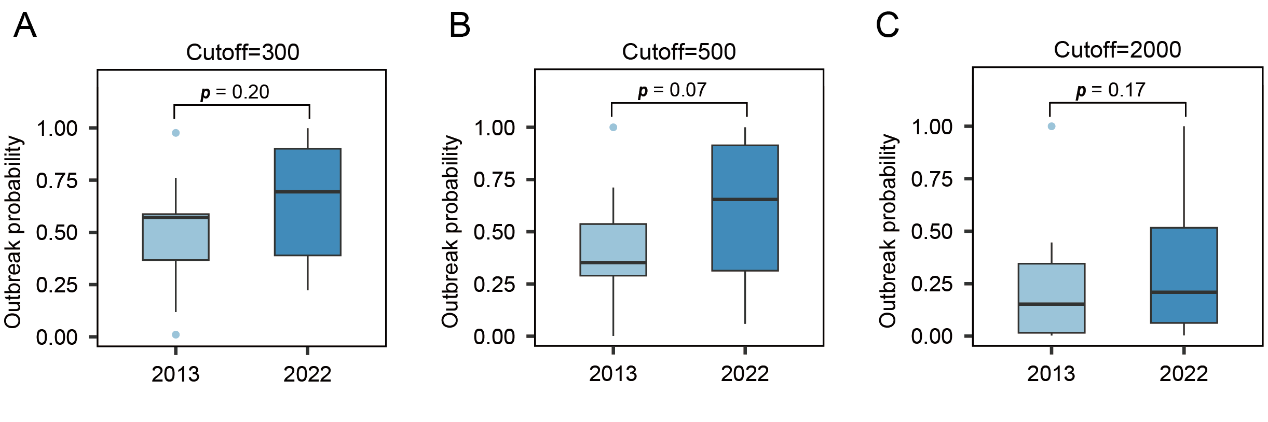


**Supplementary figure 15. Comparison of the probability of mpox outbreaks between the historical years and 2022 under different cutoff values worldwide.** (**A**) Comparison of the probability of mpox outbreaks between 2013 (n=16) and 2022 (n=32) (An outbreak is defined if the number of mpox cases exceeds 300). Each dot on the graph represents a country, p values were calculated by the Wilcoxon rank-sum test. (**B**) Comparison of the probability of mpox outbreaks between 2013 (n=16) and 2022 (n=32) (An outbreak is defined if the number of mpox cases exceeds 500). Each dot on the graph represents a country, p values were calculated by the Wilcoxon rank-sum test. (**C**) Comparison of the probability of mpox outbreaks between 2013 (n=16) and 2022 (n=32) (An outbreak is defined if the number of mpox cases exceeds 2000). Each dot on the graph represents a country, P were calculated by the Wilcoxon rank-sum test.

# Supplementary Tables

**Supplementary table 1. Study area included in the analysis**

| Country/Region (geographical scale) | Study area included |
| --- | --- |
| United States (state level) | Alabama, Alaska, Arizona, Arkansas, California, Colorado, Connecticut, Delaware, Florida, Georgia, Hawaii, Idaho, Illinois, Indiana, Iowa, Kansas, Kentucky, Louisiana, Maine, Maryland, Massachusetts, Michigan, Minnesota, Mississippi, Missouri, Montana, Nebraska, Nevada, New Hampshire, New Jersey, New Mexico, New York, North Carolina, North Dakota, Ohio, Oklahoma, Oregon, Pennsylvania, Rhode Island, South Carolina, South Dakota, Tennessee, Texas, Utah, Vermont, Virginia, Washington, West Virginia, Wisconsin, Wyoming |
| England (UTLA level) | Hartlepool, Middlesbrough, Redcar and Cleveland, Stockton-on-Tees, Darlington, Halton, Warrington, Blackburn with Darwen, Blackpool, Kingston upon Hull, East Riding of Yorkshire, North East Lincolnshire, North Lincolnshire, York, Derby, Leicester, Rutland, Nottingham, Herefordshire, Telford and Wrekin, Stoke-on-Trent, Bath and North East Somerset, Bristol, North Somerset, South Gloucestershire, Plymouth, Torbay, Swindon, Peterborough, Luton, Southend-on-Sea, Thurrock, Medway, Bracknell Forest, West Berkshire, Reading, Slough, Windsor and Maidenhead, Wokingham, Milton Keynes, Brighton and Hove, Portsmouth, Southampton, Isle of Wight, County Durham, Cheshire East, Cheshire West and Chester, Shropshire, Wiltshire, Bedford, Central Bedfordshire, Northumberland, Bournemouth, Christchurch and Poole, Dorset, Buckinghamshire, North Northamptonshire, West Northamptonshire, Bolton, Bury, Manchester, Oldham, Rochdale, Salford, Stockport, Tameside, Trafford, Wigan, Knowsley, Liverpool, St. Helens, Sefton, Wirral, Barnsley, Doncaster, Rotherham, Sheffield, Newcastle upon Tyne, North Tyneside, South Tyneside, Sunderland, Birmingham, Coventry, Dudley, Sandwell, Solihull, Walsall, Wolverhampton, Bradford, Calderdale, Kirklees, Leeds, Wakefield, Gateshead, Barking and Dagenham, Barnet, Bexley, Brent, Bromley, Camden, Croydon, Ealing, Enfield, Greenwich, Hammersmith and Fulham, Haringey, Harrow, Havering, Hillingdon, Hounslow, Islington, Kensington and Chelsea, Kingston upon Thames, Lambeth, Lewisham, Merton, Newham, Redbridge, Richmond upon Thames, Southwark, Sutton, Tower Hamlets, Waltham Forest, Wandsworth |
| Brazil (state level) | Acre, Rondônia, Mato Grosso, Mato Grosso do Sul, Paraná, Santa Catarina, Rio Grande do Sul, São Paulo, Rio de Janeiro, Espírito Santo, Minas Gerais, Distrito Federal, Goiás, Bahia, Tocantins, Piauí, Maranhão, Ceará, Rio Grande do Norte, Paraíba, Pernambuco, Alagoas, Sergipe, Pará, Amazonas, Roraima, Amapá |
| World (country level) | Argentina, Australia, Austria, Belgium, Brazil, Canada, Switzerland, Chile, Colombia, Germany, Denmark, Spain, France, United Kingdom, Hungary, India, Ireland, Italy, Japan, South Korea, Mexico, Malaysia, Netherlands, Peru, Poland, Portugal, Romania, Russia, Sweden, Turkey, United States, South Africa |

**Supplementary table 2**. **The data sources for the United States**

| **Variable** | **Definition** | **Date** | **Source** |
| --- | --- | --- | --- |
| Mpox cases | The cumulative number of mpox reported cases by December 28th, 2022. | 2022 | <https://www.cdc.gov/poxvirus/monkeypox/response/2022/us-map.html> |
|  | The cumulative number of mpox reported cases by July 23rd, 2022. | 2022 | <https://www.cdc.gov/poxvirus/monkeypox/response/2022/us-map.html> |
| Education level | The proportion of individuals with a Bachelor's degree or higher. | 2023 | <https://worldpopulationreview.com/state-rankings/educational-attainment-by-state> |
| Urbanization rate | The proportion of urban population. | 2020 | <https://www.visualcapitalist.com/sp/mapping-us-urbanization-by-state/> |
|  |  | 2010 | <https://www.icip.iastate.edu/tables/population/urban-pct-states> |
| Population density | The number of people per square mile | 2020 | <https://www.census.gov/data/tables/time-series/dec/density-data-text.html> |
|  |  | 2010 | <https://www.census.gov/data/tables/time-series/dec/density-data-text.html> |
| Proportion of LGBT individuals | The estimated percentages of adults age 18 and older who identify as LGBT. | 2020 | <https://williamsinstitute.law.ucla.edu/publications/adult-lgbt-pop-us/> |
|  |  | 2012 | https://news.gallup.com/poll/203513/vermont-leads-states-lgbt-identification.aspx |
| Smallpox vaccination coverage for 0-59 years | The coverage of smallpox vaccination for the age group of 0-59. | 2022 | estimation based on Taube J C, Rest E C, Lloyd-Smith J O, *et al*.[1] |
| Smallpox vaccination coverage for all ages | The coverage of smallpox vaccination for all age groups. | 2022 | Taube J C, Rest E C, Lloyd-Smith J O, *et al*.[1] |
|  |  | 2010 | estimation based on Taube J C, Rest E C, Lloyd-Smith J O, *et al*.[1] |
| Public educational spending | Public educational spending total average spent per pupil | 2021 | https://educationdata.org/public-education-spending-statistics |
| Public budget expenditure | Total state expenditures (in millions) | 2022 | https://www.kff.org/other/state-indicator/total-state-spending/?currentTimeframe=0&sortModel=%7B%22colId%22:%22Location%22,%22sort%22:%22asc%22%7D |
| GDP | Gross domestic product | 2022 | https://apps.bea.gov/iTable/ |
|  |  | 2012 |  |
| GDP per capita | Gross domestic product per capita (current US$) | 2022 | https://apps.bea.gov/iTable/ |
| Population growth | The exponential rate of growth of midyear population from year 2020 to 2022, expressed as a percentage. | 2022 | https://data.ers.usda.gov/reports.aspx?ID=17827 |
| Changes in mobility | Six categorized places change compared to baseline days (a normal value for that day of the week, the median value from the 5‑week period Jan 3 - Feb 6, 2020). | 2022 | https://www.google.com/covid19/mobility/ |
| Air transport passengers | Air passengers carried by airport in the state. | 2022 | https://www.bts.dot.gov/browse-statistical-products-and-data/state-transportation-statistics/us-airline-traffic-airport |
| Nighttime light index | An extended time-series (2000-2018) of global NPP-VIIRS-like nighttime light data | 2022 | https://dataverse.harvard.edu/dataset.xhtml?persistentId=doi%3A10.7910%2FDVN%2FYGIVCD&version=&q=&fileTypeGroupFacet=&fileAccess=&fileSortField=date&tagPresort=false |

**Supplementary table 3. The data sources for England**

| **Variable** | **Definition** | **Date** | **Source** |
| --- | --- | --- | --- |
| Mpox cases | The cumulative number of mpox reported cases by December 20th, 2022. | 2022 | <https://www.gov.uk/government/publications/monkeypox-outbreak-epidemiological-overview> |
| Education level | The proportion of highest level of qualification at level 4+. | 2021 | <https://www.ons.gov.uk/datasets/TS067/editions/2021/versions/3> |
|  |  | 2010 | <https://www.nomisweb.co.uk/census/2011/ks501ew> |
| Urbanization rate | The proportion of urban population. | 2010 | [https://lginform.UTLA.gov.uk/dataAndReports/search?after=2019-12-01&text=urban+population](https://lginform.local.gov.uk/dataAndReports/search?after=2019-12-01&text=urban+population) |
| Population density | The number of people per square mile | 2021 | <https://www.ons.gov.uk/peoplepopulationandcommunity/> |
| Proportion of LGBT individuals | The estimated percentages of adults age 18 and older who identify as LGBT. | 2014-2022 | <https://www.ons.gov.uk/peoplepopulationandcommunity/culturalidentity/> |
| Smallpox vaccination coverage for 0-59 years | The coverage of smallpox vaccination for the age group of 0-59. | 2022 | estimation based on Taube J C, Rest E C, Lloyd-Smith J O, *et al*.[1] |
| Smallpox vaccination coverage for all ages | The coverage of smallpox vaccination for all age groups. | 2022 | estimation based on Taube J C, Rest E C, Lloyd-Smith J O, *et al*.[1] |
|  |  | 2010 |  |
| Public educational spending | Local authority and school expenditure | 2022 | https://explore-education-statistics.service.gov.uk/find-statistics/la-and-school-expenditure/2021-22 |
| Public budget expenditure | Local authority expenditures | 2022 | https://www.gov.uk/government/statistics/local-authority-revenue-expenditure-and-financing-england-2021-to-2022-individual-local-authority-data-outturn |
| GDP | Gross domestic product | 2022 | https://www.ons.gov.uk/datasets/gdp-by-local-authority/editions/time-series/versions/2#toc |
| GDP per capita | Gross domestic product per capita | 2022 | https://www.ons.gov.uk/datasets/gdp-by-local-authority/editions/time-series/versions/2#toc |
| Population growth | The exponential rate of growth of midyear population from year 2011 to 2022, expressed as a percentage. | 2022 | https://www.gov.uk/government/statistics/statistical-digest-of-rural-england |
| Changes in mobility | Six categorized places change compared to baseline days (a normal value for that day of the week, the median value from the 5‑week period Jan 3 - Feb 6, 2020). | 2022 | https://www.google.com/covid19/mobility/ |
| Nighttime light index | An extended time-series (2000-2018) of global NPP-VIIRS-like nighttime light data | 2022 | https://dataverse.harvard.edu/dataset.xhtml?persistentId=doi%3A10.7910%2FDVN%2FYGIVCD&version=&q=&fileTypeGroupFacet=&fileAccess=&fileSortField=date&tagPresort=false |

**Supplementary table 4. The data sources for Brazil.**

| **Variable** | **Definition** | **Date** | **Source** |
| --- | --- | --- | --- |
| Mpox cases | The cumulative number of mpox reported cases by June 1st, 2023. | 2022 | <https://www.gov.br/saude/pt-br/centrais-de-conteudo/publicacoes/> |
| Education level | Proportion of population who have completed tertiary education or equivalent. | 2022 | https://sidra.ibge.gov.br/tabela/5919 |
|  |  | 2010 | https://pt.wikipedia.org/wiki/Lista_de_unidades_federativas_do_Brasil_por_porcentagem_de_pessoas_com_n%C3%ADvel_superior_completo#cite_note-IBGE_2010-1 |
| Urbanization rate | The proportion of urban population. | 2010 | <https://en.wikipedia.org/wiki/List> of Brazilian federative units by urbanization rate |
| Population density | per square kilometer of inhabitants | 2022 | https://censo2022.ibge.gov.br/panorama/mapas.html?localidade=BR&tema=1 |
|  |  | 2014 | https://www.worldatlas.com/articles/brazilian-states-by-population.html |
| Proportion of LGBT individuals | Percentage of adults who self-identify as lesbians, gay men, or bisexuals. | 2019 | [https://www.ibge.gov.br/estatisticas/sociais/saude/](https://www.ibge.gov.br/estatisticas/sociais/saude/9160-pesquisa-nacional-de-saude.html?=&t=downloads) |
| Smallpox vaccination coverage for 0-59 years | The coverage of smallpox vaccination for the age group of 0-59. | 2022 | estimation based on Taube J C, Rest E C, Lloyd-Smith J O, *et al*.[1] |
| Smallpox vaccination coverage for all ages | The coverage of smallpox vaccination for all age groups. | 2022 | Taube J C, Rest E C, Lloyd-Smith J O, *et al*.[1] |
|  |  | 2010 | estimation based on Taube J C, Rest E C, Lloyd-Smith J O, *et al*.[1] |
| Expenditure for education | Public educational spending | 2022 | https://portaldatransparencia.gov.br/funcoes/12-educacao?ano=2022 |
|  |  | 2019 |  |
| GDP | Gross domestic product | 2021 | https://www.ibge.gov.br/en/statistics/economic/national-accounts/19567-gross-domestic-product-of-municipalities.html?=&t=resultados |
| GDP per capita | Gross domestic product per capita | 2021 | https://biblioteca.ibge.gov.br/visualizacao/livros/liv102045_informativo.pdf |
| Population growth | The exponential rate of growth of midyear population from year 2021 to 2022, expressed as a percentage. | 2022 | https://censo2022.ibge.gov.br/panorama/indicadores.html |
| Changes in mobility | Six categorized places change compared to baseline days (a normal value for that day of the week, the median value from the 5‑week period Jan 3 - Feb 6, 2020). | 2022 | https://www.google.com/covid19/mobility/ |
| Air transport passengers | The total number of tickets sold between the departure and destination cities. | 2019 | https://www.ibge.gov.br/geociencias/organizacao-do-territorio/redes-e-fluxos-geograficos/15797-ligacoes-aereas.html |
| Nighttime light index | An extended time-series (2000-2018) of global NPP-VIIRS-like nighttime light data | 2022 | https://dataverse.harvard.edu/dataset.xhtml?persistentId=doi%3A10.7910%2FDVN%2FYGIVCD&version=&q=&fileTypeGroupFacet=&fileAccess=&fileSortField=date&tagPresort=false |

**Supplementary table 5. The data sources for globe.**

| **Variable** | **Definition** | **Date** | **Source** |
| --- | --- | --- | --- |
| Mpox cases | The cumulative number of mpox reported cases until December 15th, 2022. | 2022 | <https://www.cdc.gov/poxvirus/monkeypox/response/2022/world-map.html> |
|  | The cumulative number of mpox reported cases until July 23, 2022. | 2022 | https://ourworldindata.org/monkeypox |
| Education level | School enrollment, tertiary (% of gross). | 2020 | <https://data.worldbank.org/indicator/SE.TER.ENRR> |
|  |  | 2013 |  |
| Urbanization rate | The proportion of urban population. | 2020 | <https://data.worldbank.org/indicator/SP.URB.TOTL.IN.ZS?view=chart> |
|  |  | 2013 |  |
| Population density | The number of people per sq. km of land area. | 2020 | <https://data.worldbank.org/indicator/EN.POP.DNST?view=chart> |
|  |  | 2013 |  |
| Proportion of LGBT individuals | Percentage of adults who self-identify as lesbians, gay men, or bisexuals. | 2020 | <https://www.ipsos.com/sites/default/files/ct/news/documents/2021-06/lgbt-pride-2021-global-survey-ipsos.pdf> |
|  |  | 2013 | <https://www.oecd-ilibrary.org/social-issues-migration-health/a-sizeable-minority-self-identifies-as-lesbian-gay-or-bisexual_e6db64cd-en>;  <https://kpatlas.unaids.org/dashboard>;  <https://www.statista.com/statistics/1378104/netherlands-share-of-population-homosexual/> |
| Smallpox vaccination coverage for 0-59 years | The coverage of smallpox vaccination for the age group of 0-59. | 2022 | estimation based on Taube J C, Rest E C, Lloyd-Smith J O, *et al*.[1] |
| Smallpox vaccination coverage for all ages | The coverage of smallpox vaccination for all age groups. | 2022 | Taube J C, Rest E C, Lloyd-Smith J O, *et al*.[1] |
|  |  | 2010 | estimation based on Taube J C, Rest E C, Lloyd-Smith J O, *et al*.[1] |
| Acceptance of homosexuality | % who say homosexuality should be accepted by society | 2019 | https://www.pewresearch.org/global/2020/06/25/global-divide-on-homosexuality-persists/ |
| Government expenditure on education | Government expenditure on education, total (% of government expenditure) | 2020 | <https://data.worldbank.org/indicator/SE.XPD.TOTL.GB.ZS?view=chart> |
| Gross national expenditure | Gross national expenditure (current US$) | 2021 | https://data.worldbank.org/indicator/NE.DAB.TOTL.CD?view=chart |
| GNI | Gross national income (current US$) | 2021 | https://data.worldbank.org/indicator/NY.GNP.MKTP.CD?view=chart |
| GDP | Gross domestic product (current US$) | 2021 | https://data.worldbank.org/indicator/NY.GDP.MKTP.CD?view=chart |
|  |  | 2013 |  |
| GDP per capita | Gross domestic product per capita (current US$) | 2021 | https://data.worldbank.org/indicator/NY.GDP.PCAP.CD?view=chart |
| Population growth | The exponential rate of growth of midyear population from year t-1 to t, expressed as a percentage. | 2021 | https://data.worldbank.org/indicator/SP.POP.GROW |
| Changes in mobility | Six categorized places change compared to baseline days (a normal value for that day of the week, the median value from the 5‑week period Jan 3 - Feb 6, 2020). | 2022 | https://www.google.com/covid19/mobility/ |
| Air transport passengers | Air passengers carried include both domestic and international aircraft passengers of air carriers registered in the country. | 2019 | https://data.worldbank.org.cn/indicator/IS.AIR.PSGR?end=2019&start=1970 |
| Nighttime light index | An extended time-series (2000-2018) of global NPP-VIIRS-like nighttime light data | 2022 | https://dataverse.harvard.edu/dataset.xhtml?persistentId=doi%3A10.7910%2FDVN%2FYGIVCD&version=&q=&fileTypeGroupFacet=&fileAccess=&fileSortField=date&tagPresort=false |

**Supplementary table 6. The cross-validation for multiple regression models in the United States, England, Brazil and worldwide.**

|  | United States | England | Brazil | World |
| --- | --- | --- | --- | --- |
| Fold 1-AUC | 1.00 | 0.89 | 1.00 | 0.95 |
| Fold 2-AUC | 0.97 | 0.97 | 0.90 | 0.80 |
| Fold 3-AUC | 0.86 | 0.87 | 0.69 | 0.75 |
| Mean AUC | 0.94 | 0.91 | 0.86 | 0.83 |

# Supplementary References

[1] J.C. Taube, E.C. Rest, J.O. Lloyd-Smith, S. Bansal, The global landscape of smallpox vaccination history and implications for current and future orthopoxvirus susceptibility: a modelling study, Lancet Infect. Dis. 23 (2023) 454–462.

[2] Y. Zhang, N. Peng, S. Yang, P. Jia, Associations between nighttime light and COVID-19 incidence and mortality in the United States, Int. J. Appl. Earth Obs. Geoinf. 112 (2020) 102855.
